# Dosimetric evaluation of respiratory gating on a 0.35‐T magnetic resonance–guided radiotherapy linac

**DOI:** 10.1002/acm2.13666

**Published:** 2022-08-10

**Authors:** John A. Charters, Yasin Abdulkadir, Dylan O'Connell, Yingli Yang, James M. Lamb

**Affiliations:** ^1^ Department of Radiation Oncology David Geffen School of Medicine at UCLA University of California Los Angeles Los Angeles California USA

**Keywords:** compressed sensing, motion management, MRI, quality assurance, respiratory gating

## Abstract

**Purpose:**

The commercial 0.35‐T magnetic resonance imaging (MRI)‐guided radiotherapy vendor ViewRay recently introduced upgraded real‐time imaging frame rates based on compressed sensing techniques. Furthermore, additional motion tracking algorithms were made available. Compressed sensing allows for increased image frame rates but may compromise image quality. To assess the impact of this upgrade on respiratory gating accuracy, we evaluated gated dose distributions pre‐ and post‐upgrade using a motion phantom and radiochromic film.

**Methods:**

Seven motion waveforms (four artificial, two patient‐derived free‐breathing, and one breath‐holding) were used to drive an MRI‐compatible motion phantom. A treatment plan was developed to deliver a 3‐cm diameter spherical dose distribution typical of a stereotactic body radiotherapy plan. Gating was performed using 4‐frames per second (fps) imaging pre‐upgrade on the “default” tracking algorithm and 8‐fps post‐upgrade using the “small mobile targets” (SMT) and “large deforming targets” (LDT) tracking algorithms. Radiochromic film was placed in a moving insert within the phantom to measure dose. The planned and delivered dose distributions were compared using the gamma index with 3%/3‐mm criteria. Dose–area histograms were produced to calculate the dose to 95% (*D*95) of the sphere planning target volume (PTV) and two simulated gross tumor volumes formed by contracting the PTV by 3 and 5 mm, respectively.

**Results:**

Gamma pass rates ranged from 18% to 93% over the 21 combinations of breathing trace and gating conditions examined. *D*95 ranged from 206 to 514 cGy. On average, the LDT algorithm yielded lower gamma and *D*95 values than the default and SMT algorithms.

**Conclusion:**

Respiratory gating at 8 fps with the new tracking algorithms provides similar gating performance to the original algorithm with 4 fps, although the LDT algorithm had lower accuracy for our non‐deformable target. This indicates that the choice of deformable image registration algorithm should be chosen deliberately based on whether the target is rigid or deforming.

## 1 INTRODUCTION

Modern techniques in external beam radiotherapy require highly accurate knowledge of real‐time anatomical motion for precise treatment delivery to the targeted tumors while sparing critical organs. Radiotherapy to the abdomen and thorax is particularly challenging due to complex respiratory motion that may exhibit inter‐ and intra‐fraction variations and baseline drifts.^1^ A multitude of 4D computed tomography (CT) and cine magnetic resonance imaging (MRI) studies have investigated the extent of tumor and organ mobility, including lung tumors ^2^ and liver tumors.^3^ Respiratory motion is predominantly responsible for these tumor movements, with lesser contributions from other physiological processes such as peristalsis and heartbeat. Respiratory motion must be taken into account in the treatment planning process in order to ensure the tumor receives the intended dose while at the same time minimizing radiation dose to nearby organs at risk. Motion management procedures are advised for tumor trajectories exceeding 5 mm, including abdominal compression, motion encompassing methods, internal target volume (ITV) methods, real‐time tracking, and respiratory gating.^4^ Respiratory gating improves dose conformity by restricting irradiation to a fixed phase of the respiratory cycle. When the target is contained in a predefined gating boundary, or window, about the desired respiratory phase, the external beam is switched on. Conversely, if a certain percentage of the target moves beyond the window, then the beam is switched off. Gating may employ external surrogates,^5^ internal surrogates,^6^ and real‐time tumor imaging using MRI^7^ or X‐ray‐based^8^, ^9^ methods. A key advantage of respiratory gating is the reduction in the volume of normal tissue irradiated compared to ITV methods. A key risk is the possibility that inaccuracies in gating may lead to tumor underdosage. Note that, there usually exists a non‐negligible latency between the gating decision by the image‐guided system, whether the target is confined to the gating window, and the physical cessation of the external beam. Latency effects together with motion inside the window will affect the dose agreement compared to a non‐gated delivery on an equivalent static target.

The purpose of our study is to examine the dosimetric accuracy of respiratory‐gated MRI‐guided radiotherapy (MRgRT) using the ViewRay MRIdian device (ViewRay, Inc., Oakwood Village, OH). The MRIdian is one of currently two commercially available MRgRT systems, and operates at a low field strength of 0.35 T.^10^, ^11^ MRIdian allows for cine MRI during treatment mode, where a low‐resolution, real‐time image series of the targeted anatomy is displayed along a single 2D slice. Deformable image registration (DIR) is used to deform a target contour so that the target is tracked in real‐time at a rate of 4 or 8 frames per second (fps). Gating performances on MRIdian have been investigated and compared to clinical protocols for a previous version of MRIdian that used Co‐60 radiation sources.^12^ Our MRIdian system recently received research upgrades to its cine imaging, so that the images can now be acquired with compressed sensing at an accelerated rate of 8 fps. Compressed sensing in MRI is a technique to reconstruct an image from incoherently undersampled *k*‐space.^13^ There are three general requirements in order to justify the application of compressed sensing.^14^ First, the desired images must have sparse representations in some transform domain. A variety of compressed sensing techniques are available because there exist many possible sparsifying transforms. Second, the aliasing artifacts from undersampling must be incoherent in the transform domain. Finally, there must be a nonlinear reconstruction to enforce sparsity and consistency with the sampled data. Limitations of compressed sensing include artifacts from accelerated acquisition, such as blurring and global ringing, as identified in a study on brain MRI.^15^ A potential downside is a signal‐to‐noise ratio (SNR) loss with increased acceleration or regularization, which is known to occur in parallel imaging.^16^

Compressed sensing is particularly valuable for dynamic imaging in MR‐gated radiotherapy. Spatiotemporal correlations in tumor targeting along a respiratory trajectory allow for reduced *k*–*t*‐space acquisition, thereby increasing the frame rate.^17^ Such approaches were shown to be equivalent to the compressed sensing MRI framework.^18^ Therefore, compressed sensing dynamic MRI allows for increased cine frame rates but introduces potential trade‐offs in SNR. Because a subset of *k*–*t* space is sampled, the image acquisition is shorter. However, the reconstruction process may take longer.

In addition to the increased frame rate updates, MRIdian has also introduced various proprietary DIR algorithms for tracking a gating target in cine imaging. The first DIR algorithm is labeled as small mobile targets (SMT), presumably intended for relatively rigid small tumors that undergo considerable spatial translations during a breathing cycle. The second DIR algorithm is called large deforming targets (LDT), which suggests an application for tumors susceptible to lung‐induced deformations. The ViewRay treatment planning system (TPS) provides a brief description of the default and SMT algorithms that is absent from the LDT algorithm. The description states that tracking is limited to a region surrounding the target, and any points beyond the region may not be tracked.

The objective of this study is to provide a dosimetric comparison of respiratory gating on ViewRay between the original default algorithm at standard frame‐rate imaging and the novel SMT and LDT algorithms with compressed sensing accelerated imaging. The ability of gated radiotherapy to correct for subject motion will be characterized by comparing a gated treatment plan on a moving phantom to a non‐gated treatment plan on a static phantom. As our gating target for tracking will be a relatively simple contour subject to one‐dimensional motion, we anticipate that the SMT algorithm will perform better than the LDT algorithm. A standard dose agreement metric will be used to quantify the results, so that other institutions may benefit from the findings obtained here. We present dose–area histograms (DAHs) of the dose distributions for more detailed information on the spatial dose agreement.

## 2 METHODS

### 2.1 ViewRay Dynamic Phantom

Our study used the ViewRay Dynamic Phantom that is intended for commercial use in MR‐gated radiotherapy. The phantom was designed in collaboration between ViewRay and CIRS (CIRS, Inc., Norfolk, VA). The phantom body, representing a thorax, is a large cylinder the axis of which lies along the superior–inferior (SI) direction, that is, parallel to the axis of the MR bore. The phantom is of approximately water‐equivalent electron density, and the body is housed by a 1‐cm acrylic shell. A small cylindrical through‐hole enables the passage of a target insert and is located on the inferior face of the phantom body.

**FIGURE 1:**
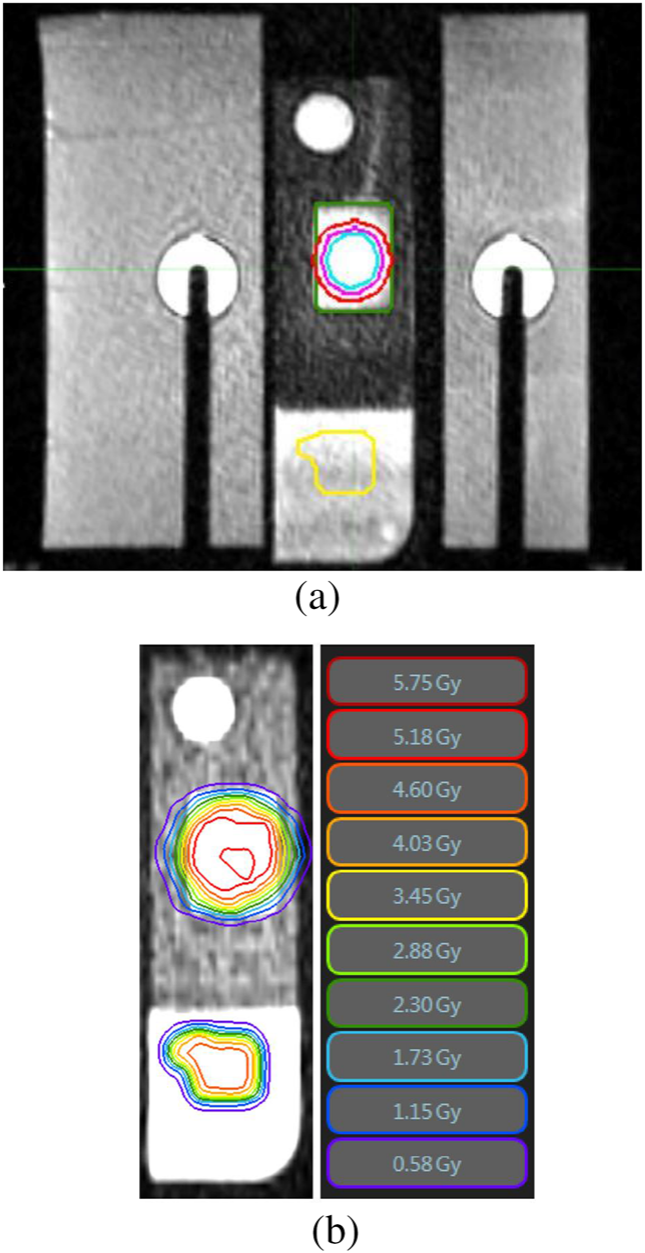
Sagittal magnetic resonance images of the ViewRay Dynamic Phantom body. The moving insert is surrounded on either side by stationary supports. The insert is attached to a rigid rod and moves vertically up and down during sagittal cine imaging based on the configured waveform: (a) dose to the reference contour (yellow) is delivered statically and is used to align the captured radiochromic dose with the planned dose distribution. The gating contour (green) is used to track the insert, and a gating window is chosen as a 3‐mm dilation of the gating contour. The spherical planning target volume (PTV) (red) is delivered with gating, and gross tumor volumes (GTVs) with 3‐(purple) and 5‐mm erosions (blue) are used in the dose–area histogram (DAH) analysis. The measured overall dimensions enclosed by the supports are 20.5 cm by 21 cm; (b) the close‐up of the moving insert with isodose lines of one fraction overlaid. The measured dimensions of the moving insert are 18.5 cm by 5 cm. The left isodose distribution is from the statically delivered reference beam that is used to extract film‐plan registration and dose scaling. At right is the test dose distribution used to evaluate gating performance.

The body and insert are composed of CIRS proprietary gels that provide high contrast to support motion tracking. The insert comprises two separate halves so that radiochromic film may be placed in‐between. See Figure 1 for an MRI of the phantom body and insert. The mean CT number of the dark region of the insert in Figure 1a is 35 HU, and the mean CT number of the brighter gel used for cine MR tracking is −60 HU.

An acrylic moving rod attaches to the insert, extends along the SI direction, and is connected to a motion actuator box at the base of the couch. Waveforms are configured in the CIRS Motion Control Software, and the reported accuracy of the resulting motion is within 0.1 mm. Crosshairs on the sides of the phantom enable in‐room laser alignment of the entire device to isocenter.

### 2.2 Motion traces

Seven waveforms were analyzed in our study. In order to simulate free breathing, two of the waveforms were cos^6^ functions with 2‐cm peak‐to‐peak amplitude. A cos^6^ function is desirable because each period has a wide trough, corresponding to a relatively longer exhalation state, followed by a briefer peak that represents an inhalation state. On the other hand, standard cosine functions have identical peaks and troughs, with an average value halfway between their range over one period. The 2‐cm peak‐to‐peak amplitude provided a realistic degree of motion with respect to our gating window. We decided to initialize the phantom position at both the exhalation (ϕ=0) and inhalation (ϕ=π/2) phases for separate cases. Periods of 4 and 6 s were chosen to represent different breathing rates.

**FIGURE 2:**
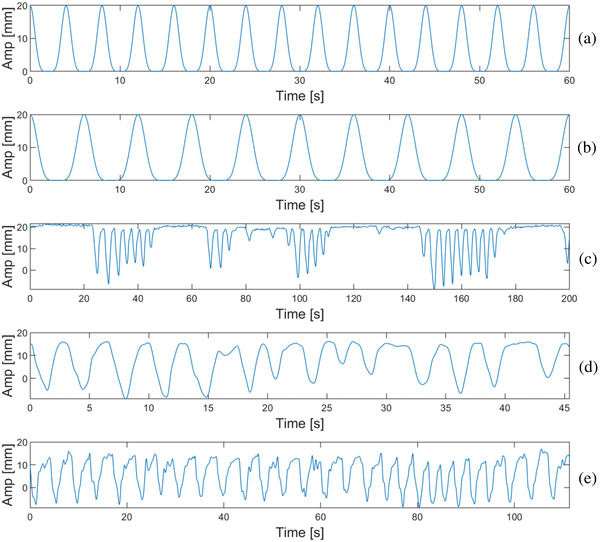
Motion traces imported into the dynamic phantom actuator. Two of the waveforms were idealistic cos^6^ functions with periods 4 s (a) and 6 s (b). The graphs as displayed here represent a ϕ=π/2 phase shift such that the inhalation state is enclosed by the gating window. To obtain the ϕ=0 phase shift with the exhalation state enclosed, we needed to briefly play the motion trace and pause at the trough. Patient 1 (c) was periodically instructed to breath‐hold, whereas patient 2 (d) and patient 3 (e) breathed freely. Patient 3 exhibited a faster breathing rate than patient 2.

Additionally, three motion traces from actual patient gating treatments were used. The motion traces were acquired by exporting tracking point locations overtime, provided by the DIR software of the MRIdian system. One trace was from a deep inspiration breath‐hold (DIBH) treatment during which the patient was instructed to perform repeated breath‐holds at a comfortable interval. The other two traces were acquired under free breathing conditions and exhibited complicated waveform patterns that were realistic manifestations of the simulated cos^6^ pattern. Phantom motion was restricted to the SI direction. Graphs of the artificial and patient motion traces are shown in Figure 2.

### 2.3 Treatment planning

A treatment plan was developed in the ViewRay TPS. A 2‐cm diameter spherical test target was irradiated with four intensity‐modulated beams. For the ease of reproducibility in aligning radiochromic film inside the phantom, we designed planar targets within a two‐dimensional coronal slice in patient‐centric coordinates. Beam angles were then chosen such that beam incidence was within 45° of normal to the film plane. The plan was normalized such that the 100% isodose surface covered 95% of the spherical target volume. Additionally, a reference beam was included in the plan at a second isocenter in the same sagittal slice, translated along the SI direction. Meanwhile, the reference target was approximately rectangular, with one corner extended asymmetrically to assist in orienting the films. The MRI in Figure 1 displays both the reference and moving contours and the expected isodose lines. A summary of the MR acquisition parameters frequently used at our institution for MRgRT is found in Table 1. Note that, spatial distortion for main‐field heterogeneity on MRIdian is a much weaker effect than gradient nonlinearity.^19^ As such, manufacturer‐provided gradient nonlinearity corrections have been applied. Dose to the reference was delivered via a parallel‐opposed beam technique, experiencing no motion and no gating. The reference beam was used for translational registration of the film to the planned dose distribution, as well as linear dose scaling per institutional film dosimetry protocol. Prescription dose was set to 750 cGy per fraction, and the planned dose was smooth across neighboring slices.

**TABLE 1:** Magnetic resonance acquisition parameters used for gradient echo imaging of the ViewRay Dynamic Phantom: (a) volumetric imaging for spatial localization, (b) cine imaging at 4 fps, and (c) cine imaging at 8 fps

Parameter	(a)	(b)	(c)
Slice thickness (mm)	1.5	4.5	4.5
In‐plane resolution (mm)	1.5 ×1.5	3.46 × 3.46	2.41 × 2.41
Matrix size (Px)	300 × 334	78 × 78	112 × 112
Echo time (ms)	1.45	1.38	1.38
Repetition time (ms)	3.37	3.26	3.26
Bandwidth (${\text{Hz}\;\text{Px}}^{-1}$)	535	556	556
Flip angle (°)	60	60	60
Averages	1	1	1

### 2.4 ViewRay dynamic imaging and gated delivery

For each delivery, the phantom target insert's motion is paused at a reference position for volumetric imaging and positioning. For the cos^6^ waveforms, the reference position was in the trough, corresponding to an exhalation state. Meanwhile, the patient breathing waveforms were initialized at the beginning of their motion trace files for consistency across scans. The phantom was then moved to isocenter and a volumetric image was acquired. The location of the phantom insert was compared to that of the planning image, and the couch was shifted as necessary.

**FIGURE 3:**
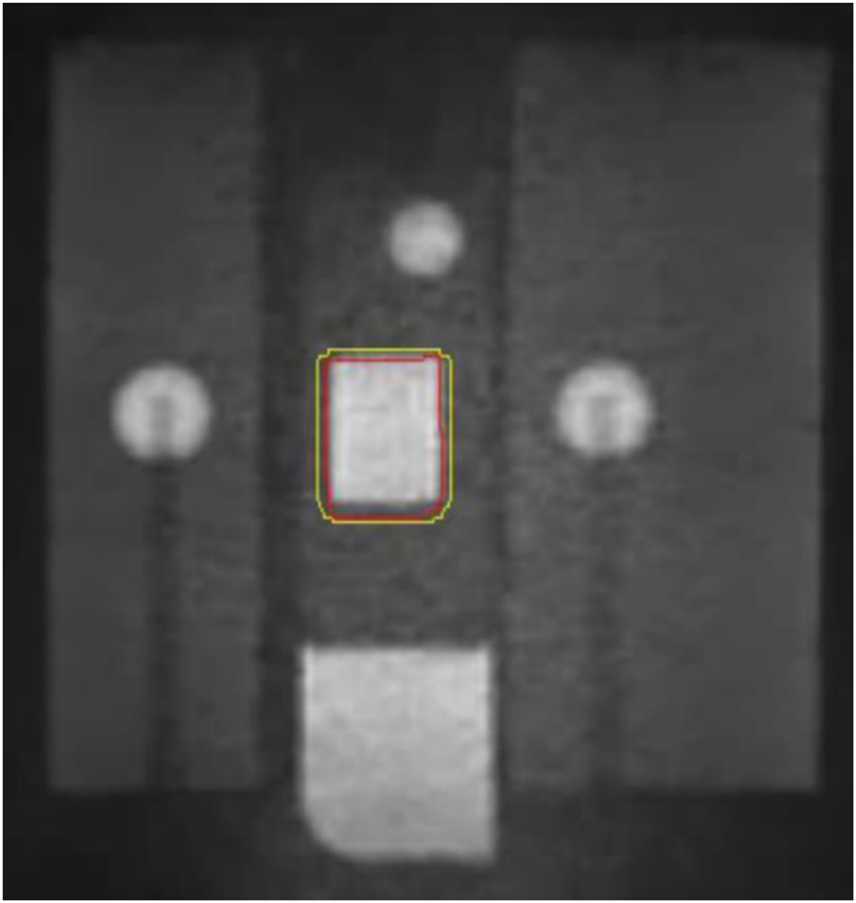
Cine magnetic resonance imaging (MRI) from 8‐fps protocol, showing the high‐contrast rectangular target in the dynamic phantom insert. Motion tracking of the target with respect to the key frame is achieved with a deformable image registration (DIR) algorithm (red contour). The gating window contour (yellow) extends 3 mm beyond the target boundary contour in the key frame. If the fraction of the target contour outside the gating window exceeds the region of interest percentage (ROI%), then the beam is toggled off.

The initial settings for gating with cine imaging include defining the tracking target and the gating window. Our convention was to choose the bright rectangle in the phantom insert as the tracking target. The dosimetric target of the treatment plan was contained within this tracking target, as shown in Figure 3. The gating window was defined as an isotropic 3‐mm expansion of the tracking target contour. The tracking target contour was deformably transferred from the treatment plan for each fraction, and slight readjustments were made as needed for better agreement with the imaged insert. Gating window margin extensions are necessary to account for small variations in tumor shape and location, as indicated in the ITV, as the tumor periodically returns to the tracking target contour. Finally, the region of interest percentage (ROI%) represents the percent of the target contour area required to leave the window before the beam is switched off. All reported data used a setting of 5% ROI. A sagittal reference slice is then required to determine a key frame for 2D tracking. We selected a sagittal slice through the center of the phantom target insert, and we maintained the same physical coordinates for all reference slices in this study for consistency.^12^ The user can choose a frame rate for displaying one sagittal slice overtime as either 4 or 8 fps, where 4 fps is the default rate and 8 fps is the recently available option that uses compressed sensing. For the cine imaging in this study, we selected the smallest field of view setting of 27 cm by 27 cm, as well as the smallest resolution setting of 0.35 cm by 0.50 cm.

The cine preview refers to the display of the 2D dynamic target before enabling treatment. Immediately after the initiation of gating, the ViewRay system acquires the following series of 2D setup images and determines a key frame by minimizing a dissimilarity measure^1^:

(1) $$F\left(A,B\right)=\;1-\frac{N\;\sum_{i}A_{i}B_{i}\;-\;\sum_{i}A_{i}\sum_{i}B_{i}}{\sqrt{N\sum_{i}A_{i}^{2}\;-\;{\left(\sum_{i}A_{i}\right)}^{2}}\sqrt{N\sum_{i}B_{i}^{2}\;-\;{\left(\sum_{i}B_{i}\right)}^{2}}}$$

where *A* is the chosen sagittal slice inside the phantom, and *B* is a setup image. The $A_{i}$ and $B_{i}$ denote respective image intensities at voxel *i*, and *N* is the total number of voxels. Usually, deformation vector field (DVF) regularization is added to the dissimilarity measure. Minimization is achieved by solving the corresponding Euler–Lagrange equations with an iterative multiscale Gauss–Seidel technique. The key frame and key frame contours act as a reference for DIR of subsequent cine frames.^20^ The ViewRay M2 software revision allows the user to select a DIR algorithm depending on the frame rate. Three different algorithms were analyzed in this study, namely, the default algorithm at 4 fps, the LDT algorithm at 8 fps, and the SMT algorithm at 8 fps. Once treatment is enabled, the ViewRay system matches the current frame to the key frame and generates DVFs to transform the key frame contours onto the current image. After the target was fully irradiated, the surrogate was paused, and the phantom was once more returned to its initial position in the motion trace. The isocenter was shifted, and the rectangular reference beam was delivered without any gating or motion.

### 2.5 GAFChromic EBT‐3 film analysis

GAFChromic EBT‐3 radiochromic film was used to capture all dose distributions.^21^, ^22^ EBT‐3 film has been tested in MRIdian real‐time imaging and has been demonstrated to be a viable method for dosimetry measurements.^23^ Rectangular segments of radiochromic film were placed in the CIRS motion phantom insert for each treatment case, and the upper and lower short ends of the film were taped down to prevent movement. Film orientation was kept consistent with respect to phantom orientation for all experiments. Before treatment plan delivery, triple‐channel film calibration was performed using the MRIdian treatment beam.^24^ Seven films were irradiated with a uniform field up to 16 Gy (including one blank), which is within the reported dynamic dose range of EBT‐3 film and was chosen to encompass the maximum dose in the treatment plan.

Film dosimetry was performed using FilmQA Pro software (Ashland, Inc., Covington, KY). An Epson flatbed scanner (Epson America, Inc., Los Alamitos, CA) was used with a glass compression plate. To eliminate the lateral response artifact, the film was placed in the same location within the flatbed scanner, with the long axis of the film cutout parallel to the scanning direction.^25^ In FilmQA Pro, the treatment plan and dose maps were overlaid for comparison. The gamma index passing rate was used to quantify dose distribution agreement.^26^ Gamma tolerances were 3%/3 mm, and pixels with less than 30% of maximum dose were suppressed, corresponding to clinical tolerances used at our institution. Dose scaling and translational shifts were applied manually, often in an iterative process, to optimize the gamma index of each statically delivered reference beam dose map compared with its treatment plan. We found that the gamma passing rate often varied by several percentage points per click of the fine translation tool. We approximated one click that corresponds to about 0.5 mm. Therefore, we can safely say that the registration is accurate to within 0.5 mm. Due to potential angular misalignments about either short end of the radiochromic film within the phantom insert, we allowed for minor lateral shifts based on the small‐angle approximation sinx≈x, 0≤x≪1, when overlaying the dose maps. Once the dose maps are registered according to the static region, we read out the gamma index of the gated spherical target. Table 2 provides a summary of this procedure for computing gamma indices.

**TABLE 2:** Procedure for computing gamma indices with radiochromic film

1.	Scan the irradiated film with triple‐channel calibration to create a planar film dose map
2.	Draw an ROI centered on the reference beam
	Use translational shifts to align the dose map to the planned dose distribution
3.	Adjust the planned dose scaling factor
4.	Repeat steps 2 and 3 to optimize the gamma passing rate within the ROI^a^
5.	Move the ROI^a^ to the target beam
6.	If necessary, perform fine translational adjustments in the direction orthogonal to motion
7.	Record the gamma index on the target beam ROI^a^

### 2.6 Analysis

To further evaluate the dosimetric agreement, we defined two hypothetical gross tumor volumes (GTVs) in MIM (MIM Software, Inc., Cleveland, OH) using morphological contractions of the sphere planning target volume (PTV) by both 3 and 5 mm (see Figure 1). As the PTV and GTV contours could not be imported into FilmQA Pro, we designed custom scripts in MATLAB (MathWorks, Natick, MA) to carry out the analysis. The planned dose from the ViewRay TPS and the contours from MIM were both exported in DICOM format. The contour data points were resampled onto the sagittal slice containing the planned reference and test beams. Next, the scanned radiochromic dose bitmaps were exported from FilmQA Pro, and the red channels were rescaled to cGy using a constant value recorded in FilmQA Pro. The film dose was registered to the planned dose by maximizing the cross‐correlation between the arrays. The dose scaling and lateral shifts determined for each case when maximizing the gamma passing rate in FilmQA Pro were then applied in our script as well. Gamma maps were computed with our own MATLAB function using equations contained in Low et al.^26^ Finally, the contours were converted to binary masks in order to create DAHs and extract the *D*95 quantities.

The dosimetric outcome of the three tracking algorithms was compared pairwise. We considered both a nonparametric Wilcoxon signed‐rank test and a parametric Student's *t*‐test with paired data between two algorithms at a time.^27^ Two‐tailed *p*‐values were calculated in MATLAB (MathWorks, Natick, MA). Our null hypothesis for the signed‐rank test was that the gamma passing rate percentage differences between any two algorithms have median zero. Our null hypothesis for the *t*‐test was that these passing rate percentage differences come from a normal distribution.

## 3 RESULTS

**FIGURE 4:**
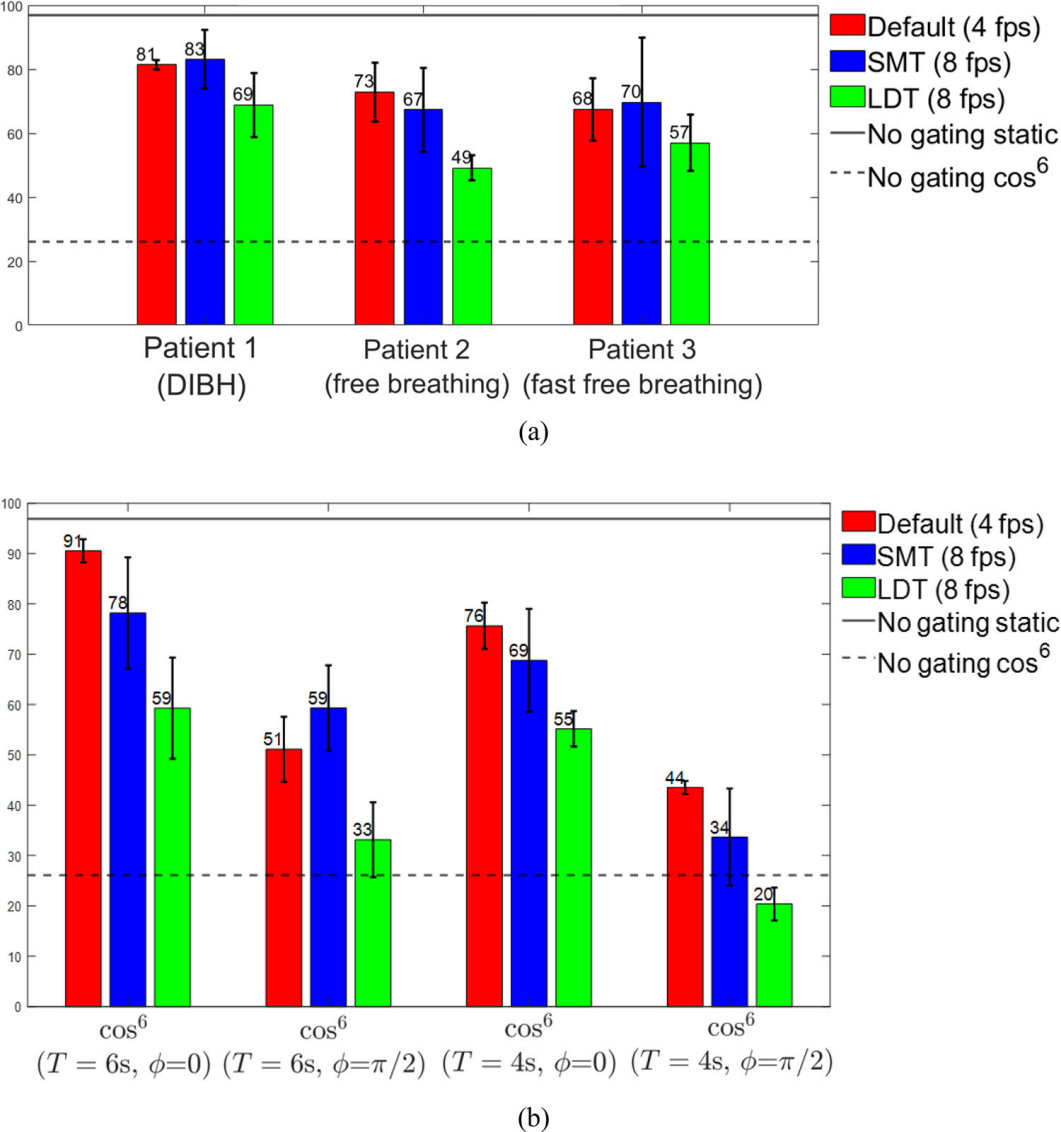
Gamma quality index comparison between the planning dose distribution and the dose distribution measured with radiochromic film. Each data point represents the mean of N=3 measurements, with error bars covering a 95% confidence interval: (a) patient waveforms. For comparison, we included gamma values for deliveries without gating on a static object (solid line) and a cos^6^ periodically moving object (dashed line); (b) ideal cos^6^ waveforms. A phase of ϕ=0 indicates that the inhalation state, or the peak of the wave, is contained inside the gating boundary. On the other hand, a phase of ϕ=π/2 indicates that the inhalation state is outside the gating window and the tracking target moves into the gating boundary upon exhalation.

The gamma passing rate percentages for the dynamic target with a gated delivery are shown in Figure 4. The measurements at 4 fps without compressed sensing were recorded prior to the ViewRay upgrades when there was no option to select a DIR algorithm. Meanwhile, the two DIR algorithms investigated at 8 fps were the LDT and the SMT. The gamma indices for all reference beams delivered statically met our clinical standard of at least 95%. Gamma pass rates ranged from 18% to 93% over the 21 combinations of breathing trace and gating conditions examined. The *D*95 ranged from 206 to 514 cGy. On average, the LDT algorithm yielded lower gamma values and lower *D*95 than the default and SMT algorithms.

**FIGURE 5:**
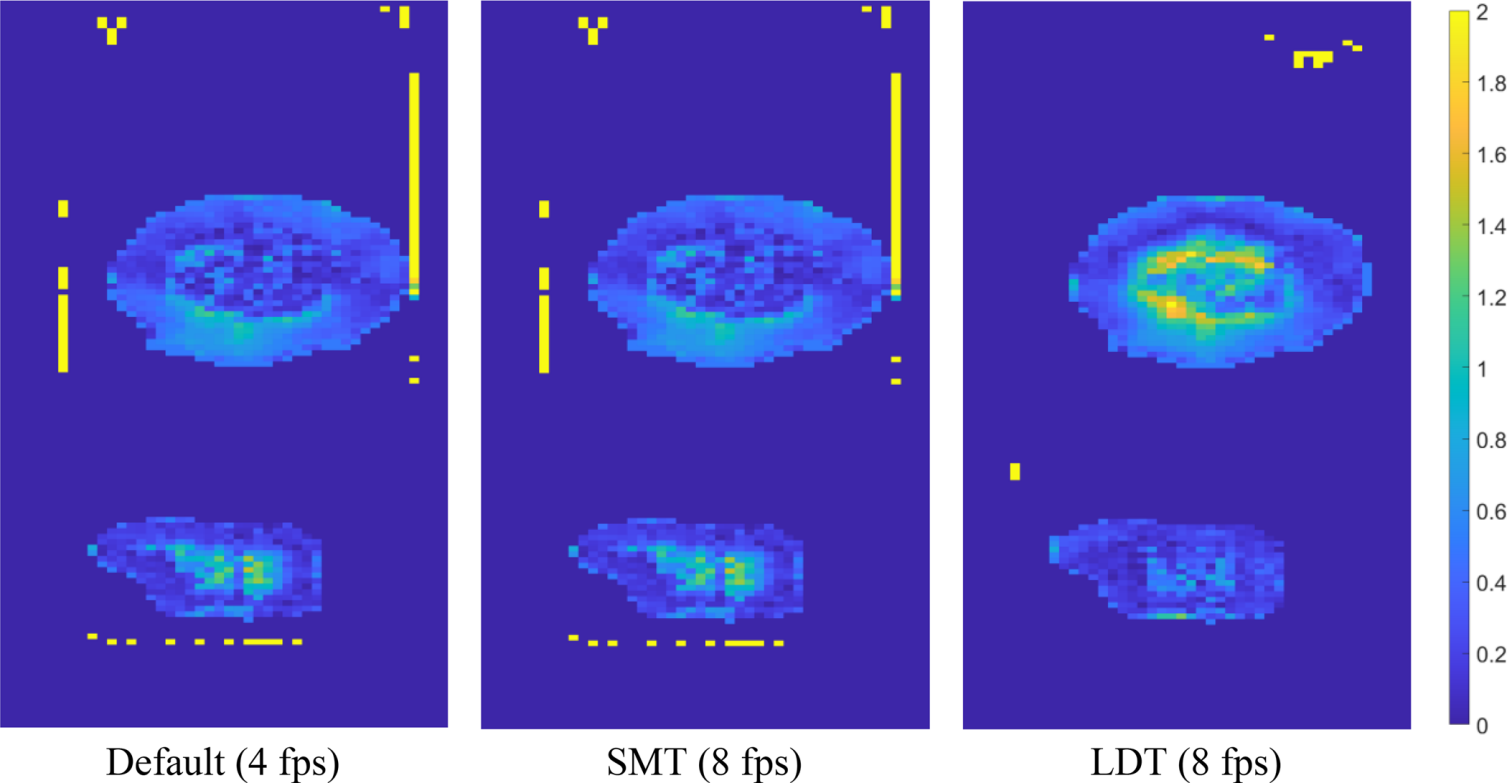
Gamma maps for a cos^6^ wave with amplitude 20 mm and period 6 s. The phase shift is ϕ=0, that is, the trough of the wave is inside the gating window, whereas the peak is outside. We chose a tolerance of 3%, a distance to agreement of 3 mm, and a minimum threshold of 30%. The computed gamma maps are global in the sense that the dose differential is normalized with respect to the maximum planned dose.

**FIGURE 6:**
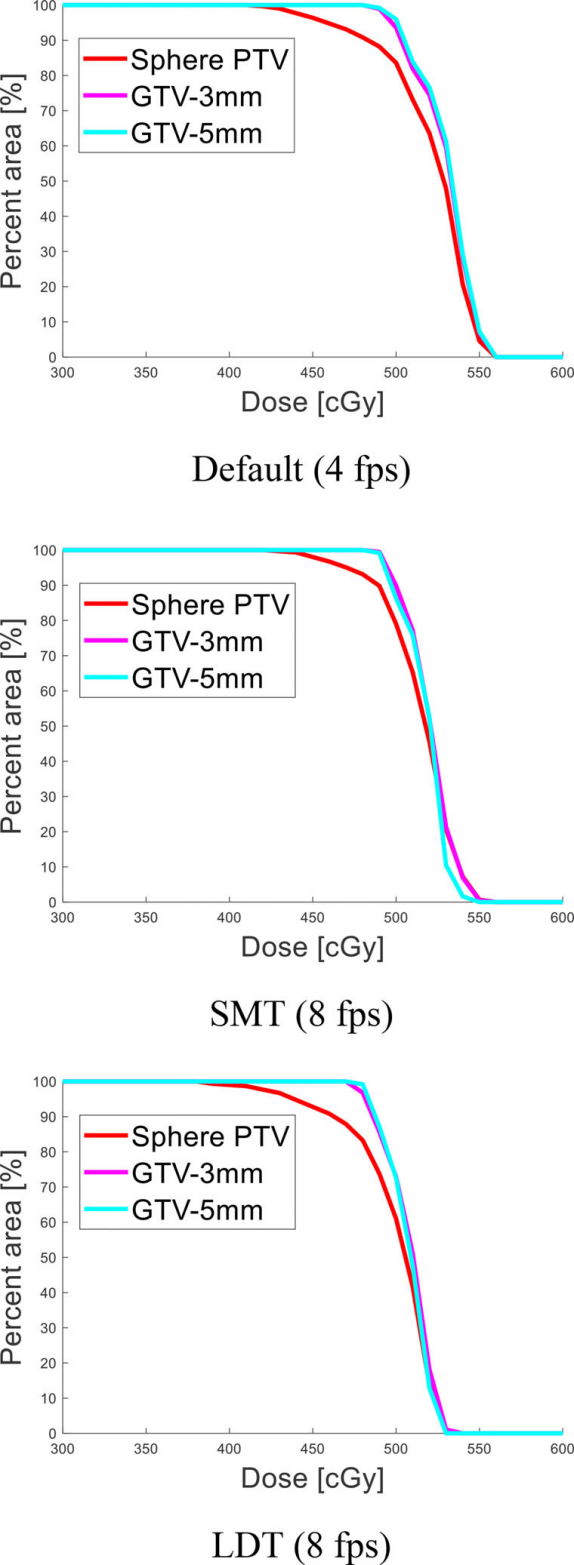
Dose–area histograms (DAHs) for a cos^6^ wave with amplitude 20 mm and period 6 s. The phase shift is ϕ=0, that is, the trough of the wave is inside the gating window, whereas the peak is outside. In order to further assess the dose compactness, we performed a morphological contraction of the original sphere planning target volume (PTV) (red) to create gross tumor volumes (GTVs) with 3‐mm (magenta) and 5‐mm (cyan) margins.

An un‐gated delivery with a moving target, the trajectory of which was a cos^6^ function with 2‐cm amplitude and 6‐s period, resulted in a gamma index of 26.1%. This measurement validates the substantial dosimetric improvement when respiratory gating is applied compared to non‐gated deliveries that do not compensate for target motion. Meanwhile, an un‐gated delivery with a static target resulted in a gamma index of 96.9%, which represents the ideal upper limit when respiratory gating perfectly compensates for motion and replicates a static delivery. These values are indicated with horizontal lines in Figure 4. The average gamma indices across all motion traces for the 4 fps, 8‐fps SMT, and 8‐fps LDT algorithms were 69%, 66%, and 49%, respectively. Gamma maps and DAHs for an example breathing pattern are shown in Figures 5 and 6, respectively. The results for every case are available as supplemental material. The *D*95 values on our PTV and GTV contours are listed in Table 3.

**TABLE 3:** Dose coverage to 95% of the test target volumes (*D*95) for the default, small mobile targets (SMT), and large deforming targets (LDT) algorithms, respectively

Waveform	Sphere PTV	GTV, 3‐mm margins	GTV, 5‐mm margins
Static	490	497	494
cos^6^ (*T* = 6 s, ϕ=0)	**459**/**470**/**439**	497/495/482	501/493/483
cos^6^ (*T* = 6 s, ϕ=π/2)	**469**/**426**/**343**	505/489/**445**	512/492/482
cos^6^ (*T* = 4 s, ϕ=0)	**457**/**432**/**416**	483/484/**461**	487/487/**472**
cos^6^ (*T* = 4 s, ϕ=π/2)	**344**/**296**/**206**	**413**/**401**/**324**	**447**/**452**/**384**
Patient 1 (breath‐hold)	**463**/**471**/**455**	504/485/499	509/482/508
Patient 2 (free breathing)	**436**/**464**/**424**	490/501/**475**	499/507/490
Patient 3 (fast free breathing)	**446**/**469**/**455**	499/505/**475**	514/512/**473**

Comparing the default and LDT algorithms resulted in a Wilcoxon *p*‐value of $8.0\;\times \;10^{-5}$ and Student's *p*‐value of $2.4\;\times \;10^{-8}$. Comparing the default with SMT resulted in a Wilcoxon *p*‐value of 0.36 and Student's *p*‐value of 0.31. Finally, comparing SMT and LDT yielded a Wilcoxon *p*‐value of $9.2\;\times \;10^{-5}$ and Student's *p*‐value of $4.2\;\times \;10^{-7}$.

**FIGURE 7:**
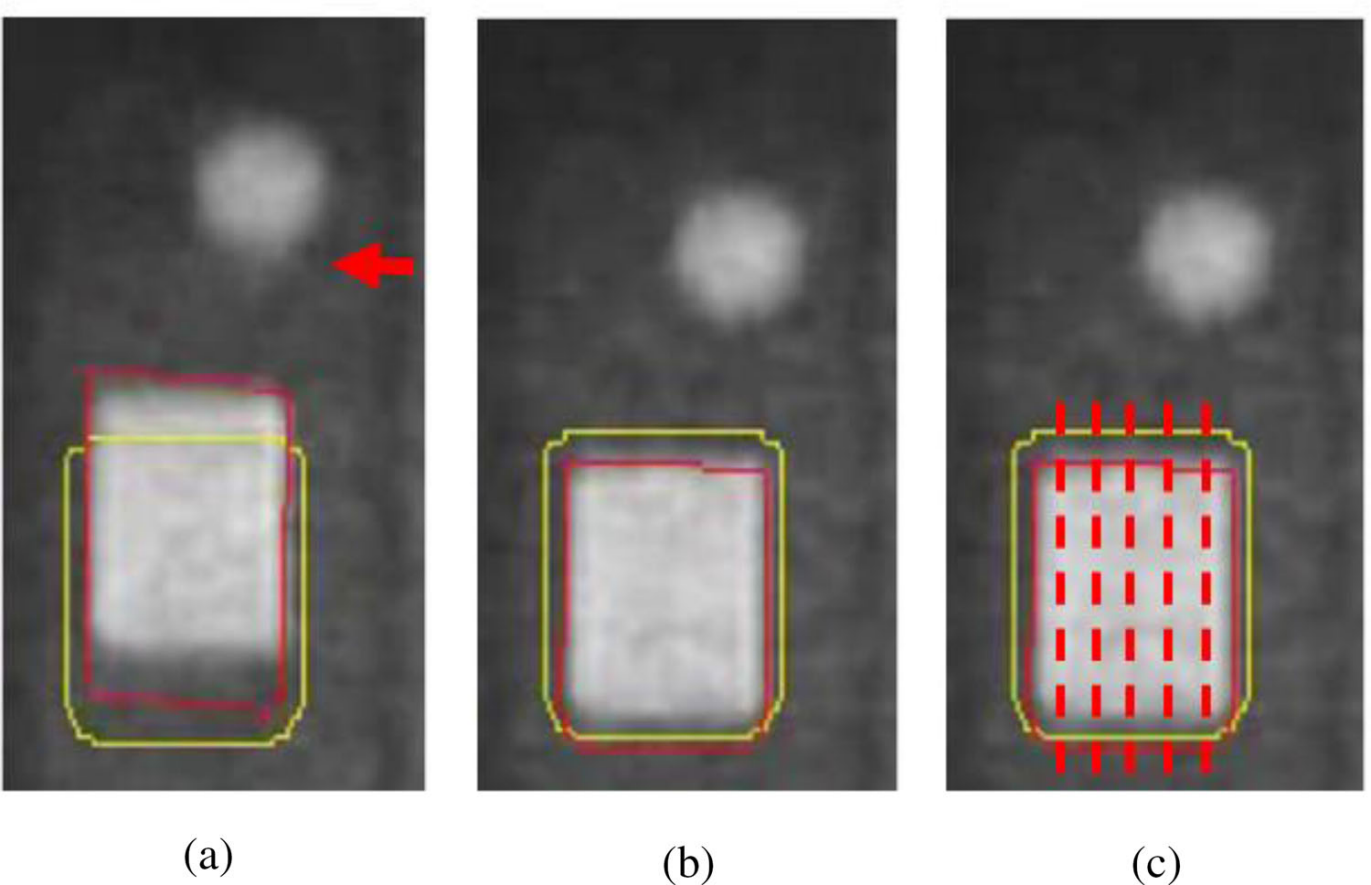
(a) Blurring visible in 8‐fps images at mid‐position of a cos^6^ trajectory (red arrow). Compare to (b), an image acquired with phantom at rest, without blurring. (c) Illustration of line profiles (dashed red lines) used to quantify blurring

**FIGURE 8:**
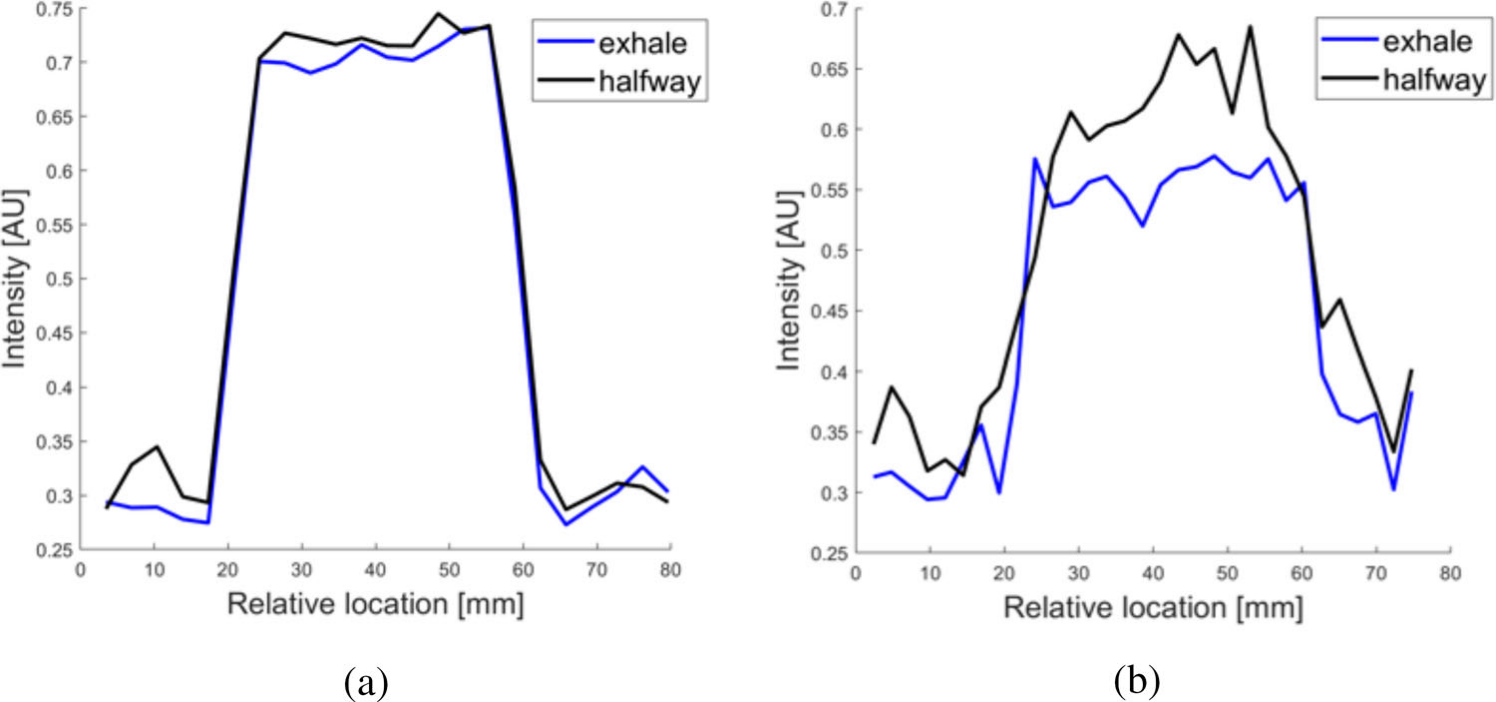
Line profiles for the high‐contrast phantom target along the direction of motion: (a) cine imaging at 4 fps. Line spread function of the target edge at the exhale and halfway breathing phases are nearly identical; (b) cine imaging at 8 fps with compressed sensing. Although more noise is present in the compressed sensing images, the line spread function is clearly shallower at the halfway breathing phase (maximal phantom motion) compared to the exhale phase (static phantom).

Latency refers to the time delay between the gating signal and the beam toggle and is measured as part of routine quality assurance (QA) at our institution. The average beam‐off latency was measured at 150.6 ms with 4 fps and 118.1 ms with 8 fps in routine QA. The average beam‐on latency for our system was measured at 318.1 ms with 4 fps and 597.4 ms with 8 fps. During these investigations, an apparent blurring was observed in the 8‐fps images (see Figure 7a). This effect was investigated using line profiles. Figure 8 shows line profiles from 4‐ and 8‐fps imaging. Line profiles are plotted for images from the trough of the cos^6^ waveform, where the phantom is at rest, and the midpoint of the waveform where the phantom speed is maximal. Line profiles pass through the superior tracking target edge and were averaged over the transverse length of the tracking target in order to reduce noise (as illustrated in Figure 7c). For the 4‐fps imaging, line profile rising edges had equal steepness in the image of the phantom at rest and in motion, indicating a lack of motion blurring. In contrast, for the 8‐fps imaging, the line profile of the phantom at rest was markedly steeper than the line profile of the phantom in motion, indicating the presence of motion blurring.

## 4 DISCUSSION

Compressed sensing is a modern development in accelerated imaging that shows promise in IGRT applications. Our study has made progress in a detailed understanding of the gating accuracy of the ViewRay MRIdian with various tracking algorithms and compressed sensing image acceleration. As the algorithms are proprietary to MRIdian, a quantification of the imaging and reconstruction latencies for each algorithm was beyond the scope of this study. Notice that for the cos^6^ waves, the gamma index is worse for a shorter period due to the increased relative significance of gating latency. As for latency differences between frame rates, it is interesting that the measured beam‐on latency increases with 8‐fps imaging, whereas the beam‐off latency decreases. These measurements are unexpected and warrant further investigation. The findings in Figure 8 suggest an increased motion blurring at 8 fps, which may be more pronounced for the LDT algorithm. A potential explanation is related to data sharing between adjacent frames in time, which could be made more precise with an accurate quantification of imaging and reconstruction latencies. It is unclear how motion blurring may affect gating latency in‐phantom measurements, or in practical clinical situations.

Although the gamma passing rates for gated beams are under 95%, the metric that we ultimately employ for successful deliveries is *D*95. Based on the results in Table 3, the GTVs with 3‐ and 5‐mm margins received within 5% of the intended test dose for the default and SMT algorithms, besides just one cos^6^ case with the exhalation phase outside the gating window. However, about half of cases using the LDT algorithm did not receive enough dose. Thus, we conclude that the LDT algorithm was unsuccessful about half of the time, whereas the default and SMT algorithms sufficiently covered the GTVs. We can also conclude that it is undesirable to have the tumor target enter the gating window upon inhalation, rather than exhalation, for fast breathing.

The motion traces from real patients provided a more realistic situation for gating. As expected, the DIBH gating protocol outperformed free breathing gating protocols. Respiratory gating with the compressed sensing LDT algorithm yielded worse dose agreement compared to that with the default algorithm at 4 fps and the compressed sensing SMT algorithm. This finding was statistically significant for both parametric and nonparametric tests. However, dose agreements between the compressed sensing SMT and the default 4‐fps algorithms were not statistically distinguishable.

Note that the 5% ROI we used in the gating setup is more restrictive than our clinical protocol of a 10% ROI for in vivo tumors. This implies that the gated treatments in our study were more restrictive in terms of the target proximity to the key frame before the beam is switched on. However, our gating target was also a simple rectangle with a relatively high contrast against the phantom insert background, and the SMT DIR algorithm is most appropriate for this scenario. In vivo tumors are more irregularly shaped and usually exhibit considerably lower contrast, which could make tumor tracking more difficult for DIR algorithms. Qualitatively, the effects of higher contrast and smaller ROI% will tend to offset each other. Another possible limitation of this study is the linear SI motion across all treatment cases. Nonlinear tumor motion, such as elliptical trajectories due to lung hysteresis,^28^ may influence the behavior of the DIR algorithm accuracy. At a significance level of 0.05, gamma indices for the default and SMT algorithms were not statistically distinguishable with a *t*‐test. However, the 8‐fps LDT algorithm yielded statistically worse gamma indices, assuming the paired differences in values belong to a normal distribution. Our phantom study demonstrated here may be used by other institutions to help evaluate high frame rate imaging on their gating protocols.

## 5 CONCLUSION

The 0.35‐T MRI‐guided linac MRIdian by ViewRay is one of two commercially available MRgRT systems. MRIdian is capable of cine MRI during treatment mode, which is critical for tumor motion tracking. Tumor motion is frequently managed by respiratory gating, where the radiation beam is switched off whenever the tumor leaves a fixed boundary. Consequently, the accuracy of motion tracking software will impact gating dosimetry performance. Compressed sensing MR is a technique to accelerate the imaging frame rate and, thereby, aims to improve tracking accuracy. Our study has made progress in a detailed understanding of gating accuracy with various tracking algorithms and with/without compressed sensing image acceleration.

Respiratory‐gated MRgRT treatments with compressed sensing imaging show comparable performance to treatments with standard frame rate imaging. In this study, three DIR tracking algorithms were evaluated, namely, the default algorithm at 4 fps, the SMT algorithm at 8 fps, and the LDT algorithm at 8 fps. This study applies for a simplistic planar geometry with an in‐plane evaluation and one‐dimensional motion along the SI direction. For more complex three‐dimensional geometries with motion in various directions, a single imaging slice may not be sufficient. The particular algorithm selected for motion tracking in the real‐time images influences gated dose distributions and should be chosen deliberately. Respiratory gating at 8 fps with the new tracking algorithms provides similar gating performance to the original algorithm with 4 fps, although the LDT algorithm had lower *D*95 agreement for our non‐deformable target. Therefore, we recommend that for a rigid PTV, the choice of DIR algorithm should be either default or SMT and not LDT. Furthermore, for fast breathing motion patterns, it is unadvised to gate on the inhalation phase of the respiratory cycle, as all *D*95 values for this case were unacceptable by our clinical standards. This study is the first to provide a detailed evaluation of the effectiveness of fast frame rate imaging on an MR‐gated radiotherapy system.

## AUTHOR CONTRIBUTIONS

John A. Charters and James M. Lamb conceptualized and designed the project. All authors contributed to gathering the data. All authors participated in manuscript drafting and final approval of the manuscript.

## CONFLICT OF INTEREST

James M. Lamb and Yingli Yang report consulting income from ViewRay Inc., outside of this work.

## Supporting information



Supplementary information

Supplementary information

Supplementary information

## Footnotes

1 This information was gathered from an internal ViewRay physics training document.
