# Supplementary material for: Dosimetric evaluation of respiratory gating on a 0.35‐T magnetic resonance–guided radiotherapy linac
Source: J Appl Clin Med Phys. 2022 Aug 10;23(9):e13666. doi: 10.1002/acm2.13666 (PMC9815517; doi:10.1002/acm2.13666)
Supplement: Supplementary file 1 — Supplementary information [file ACM2-23-e13666-s001.docx]

|  | Default (4 fps) | SMT (8 fps) | LDT (8 fps) |
| --- | --- | --- | --- |
| 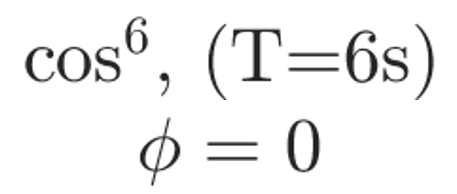 | 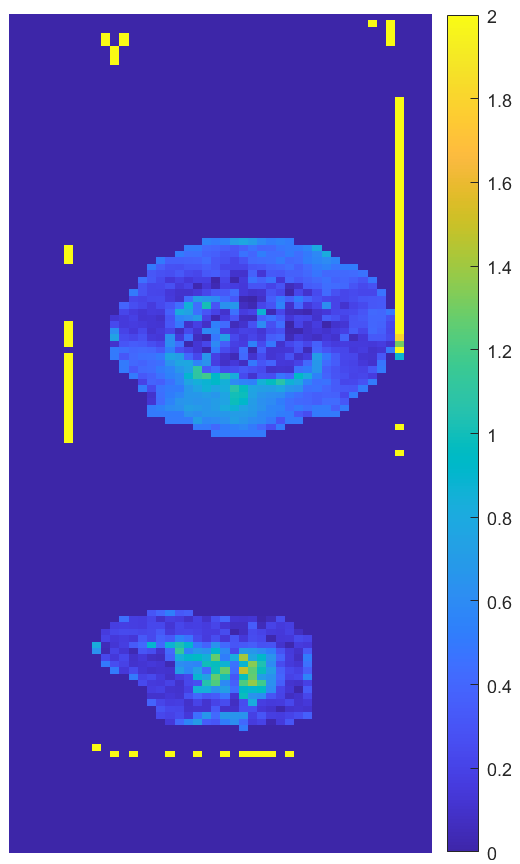 | 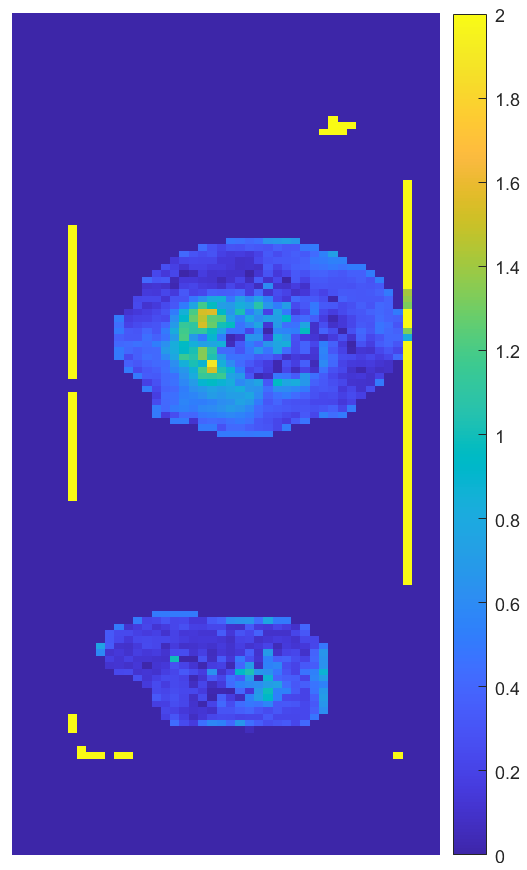 | 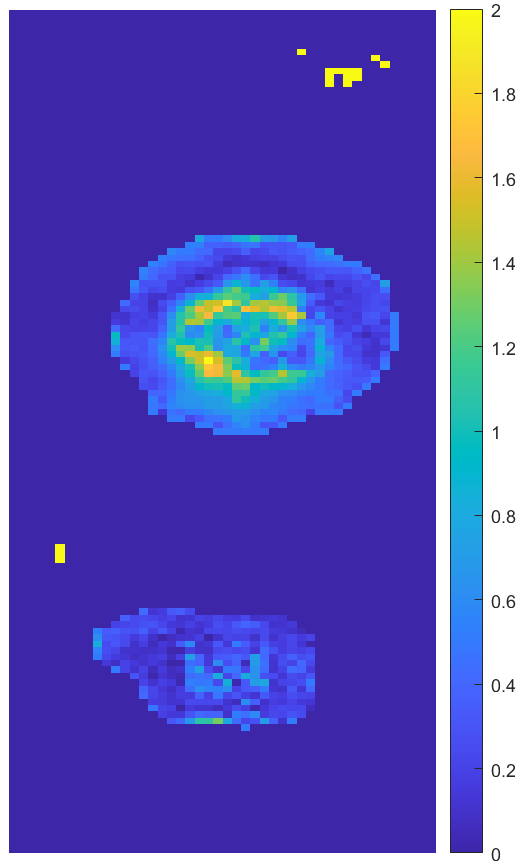 |
| 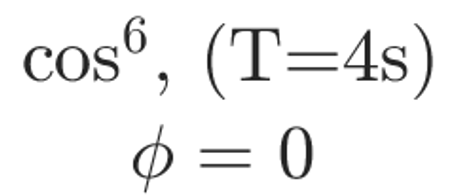 | 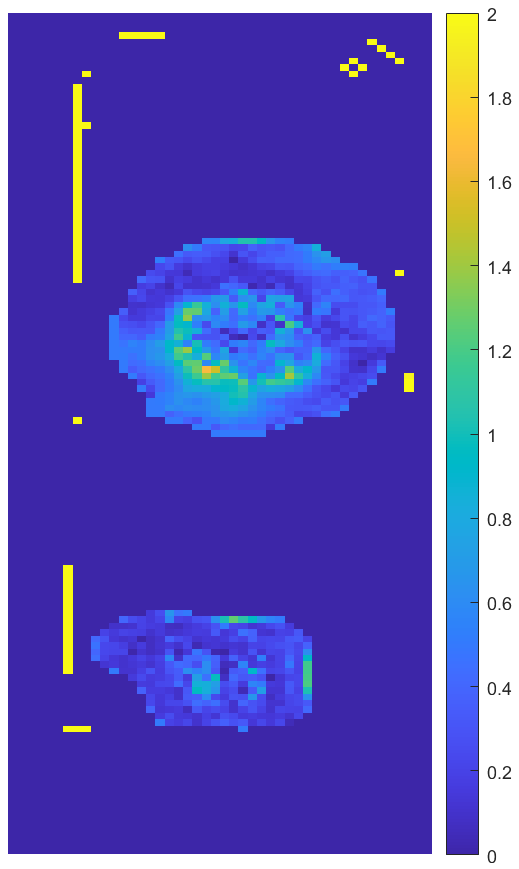 | 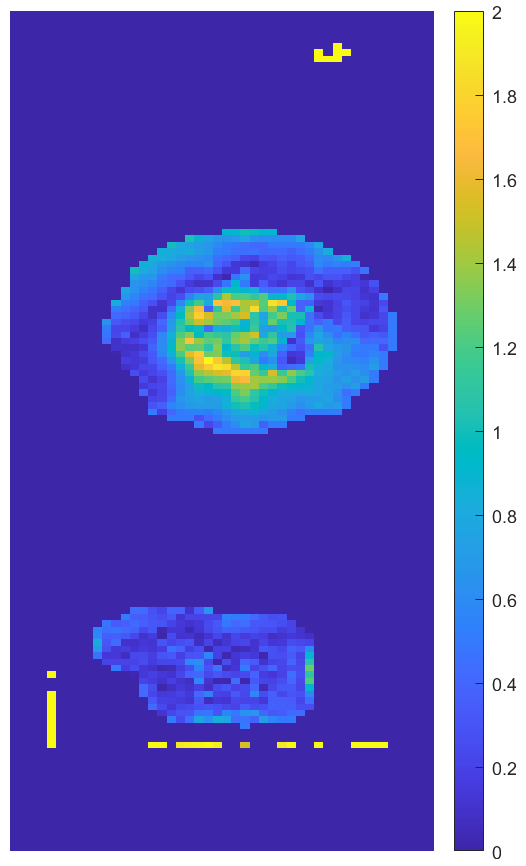 | 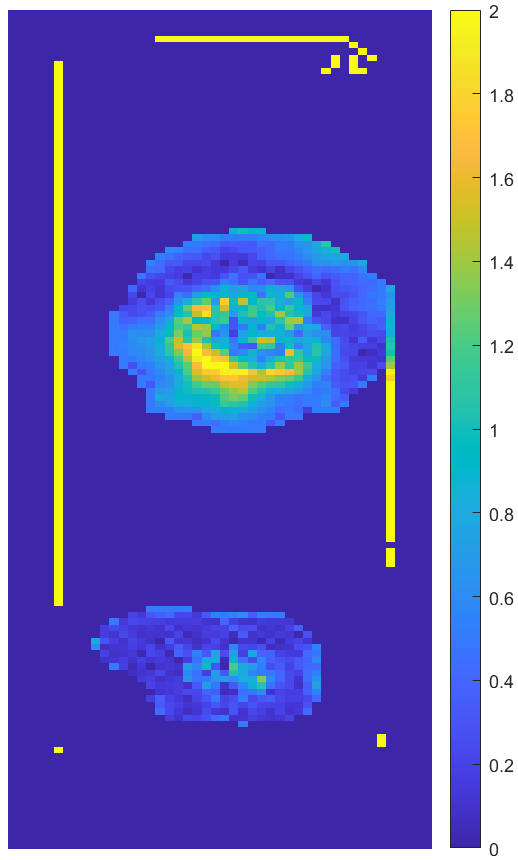 |
| 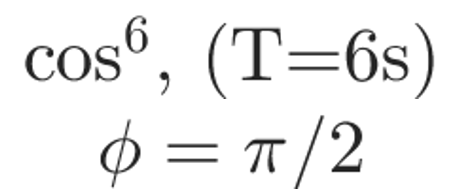 | 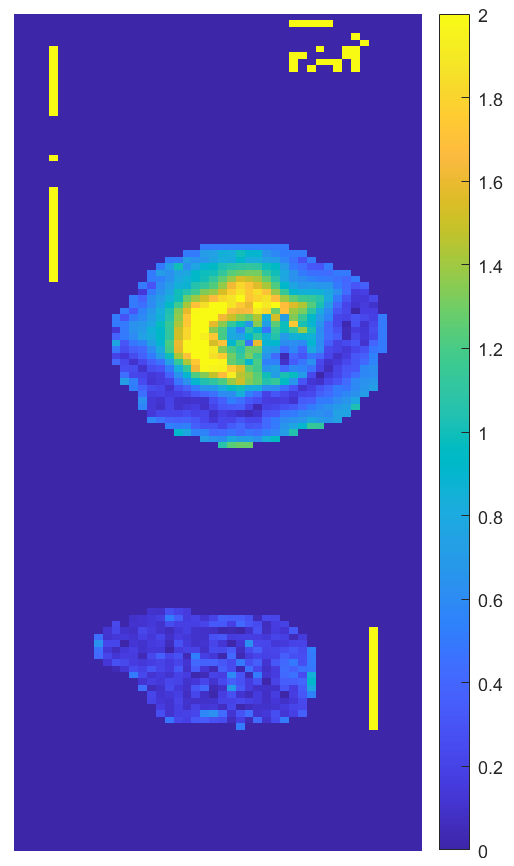 | 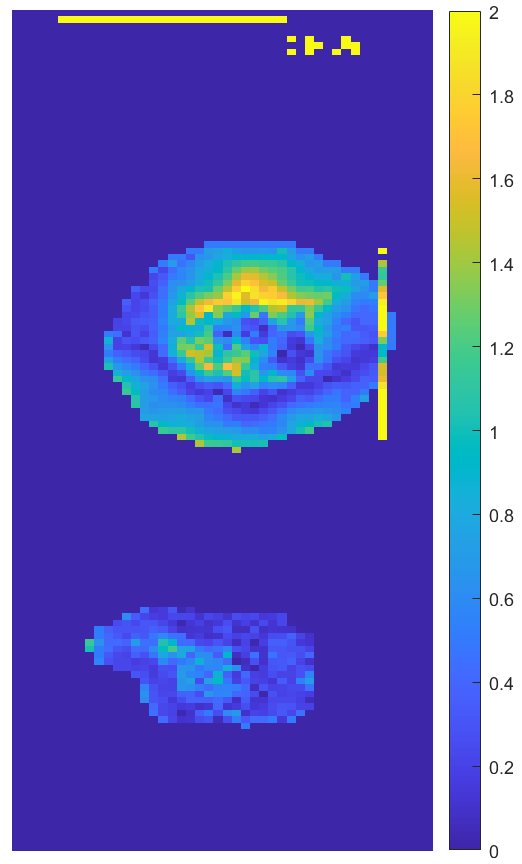 | 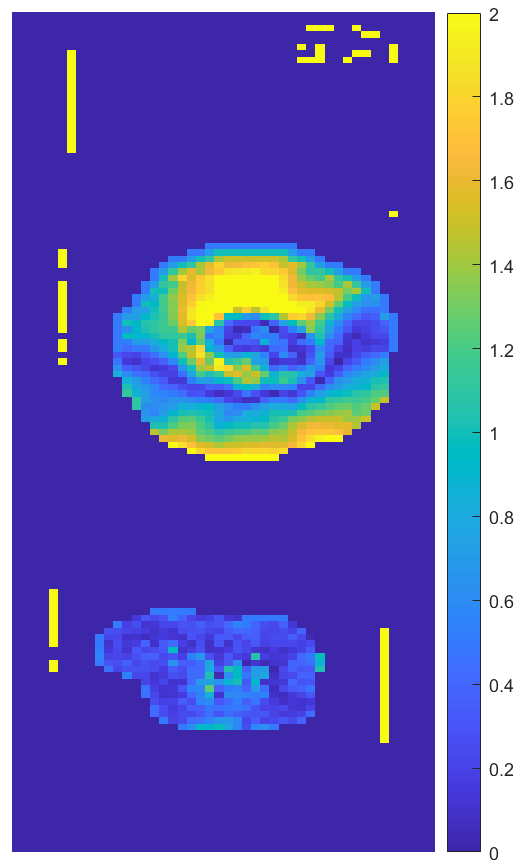 |
| 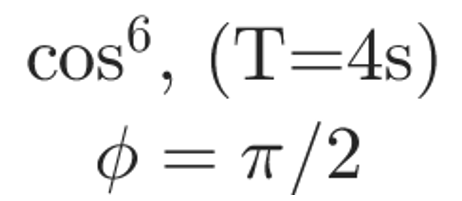 | 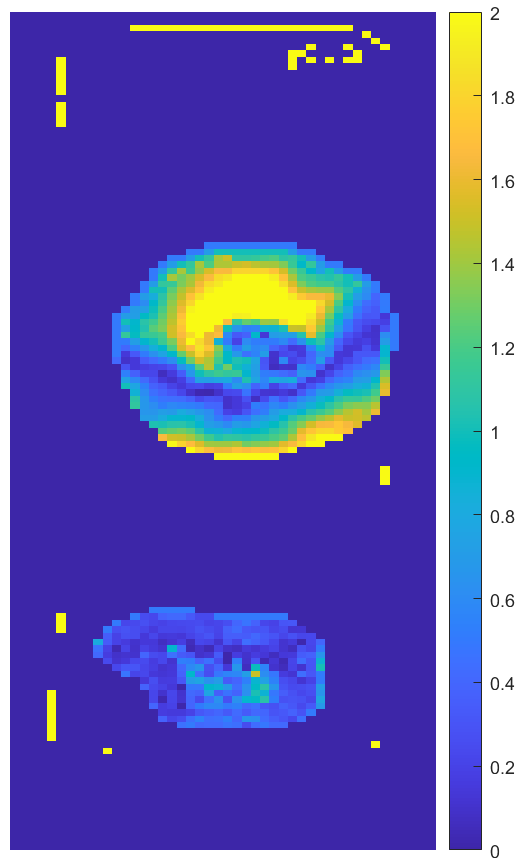 | 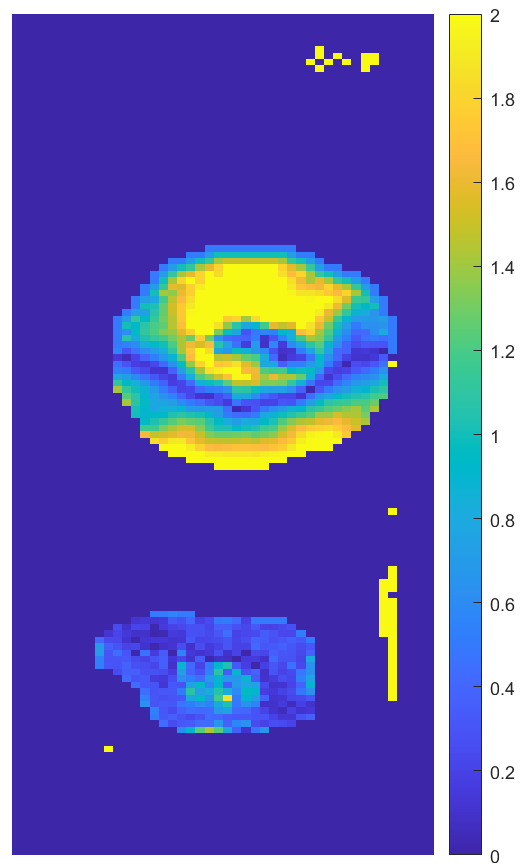 | 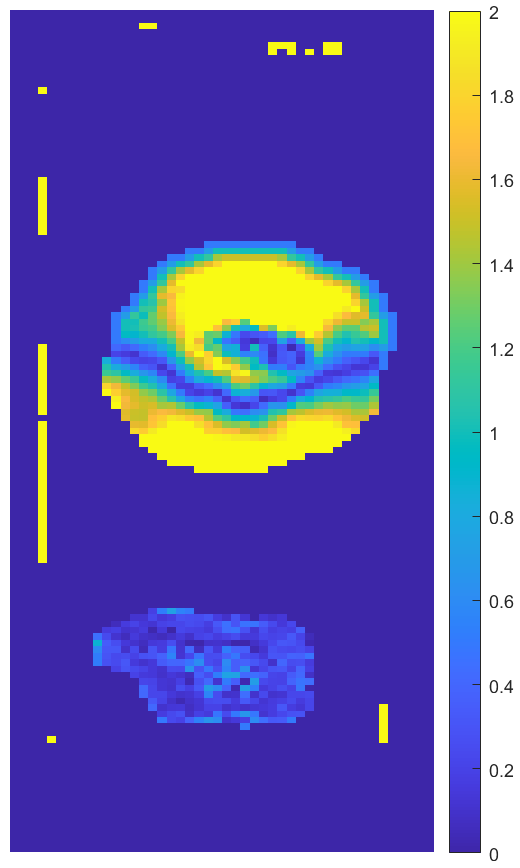 |
| 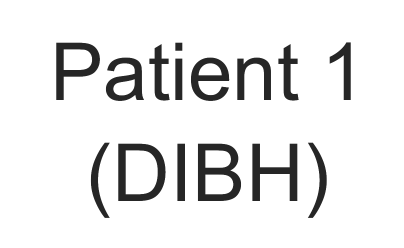 | 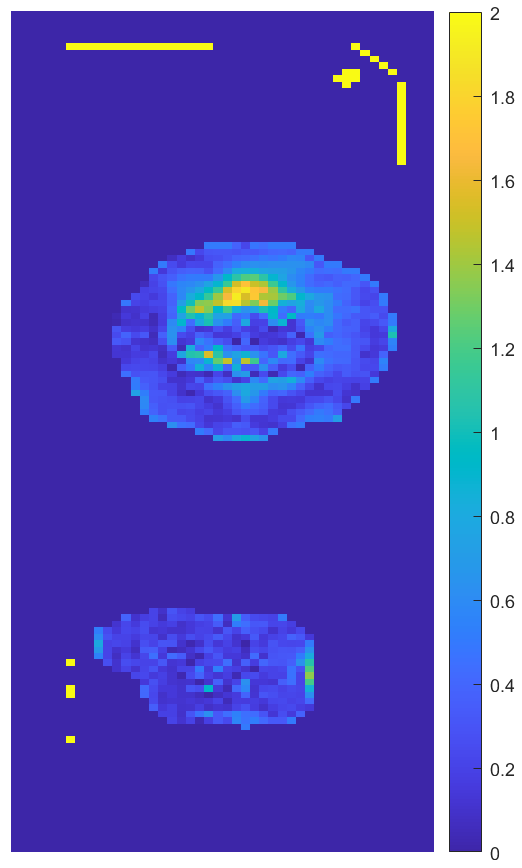 | 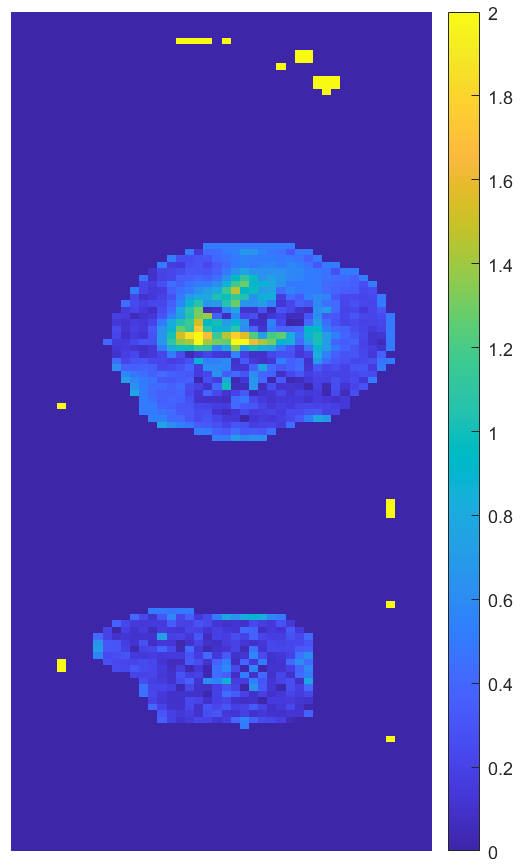 | 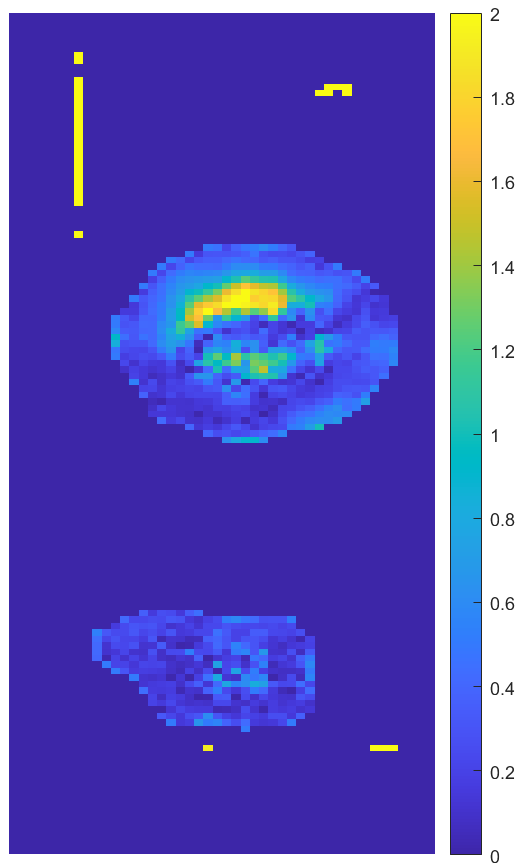 |
| 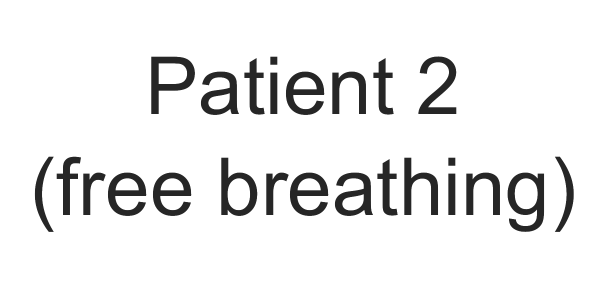 | 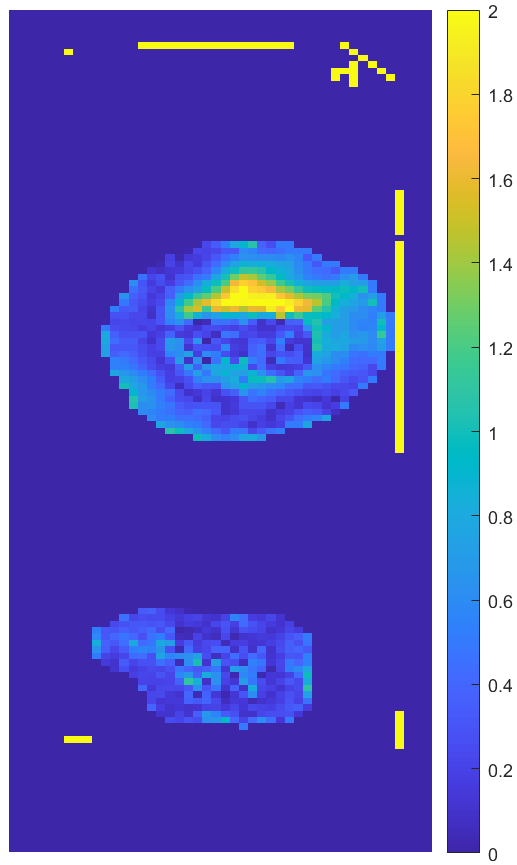 | 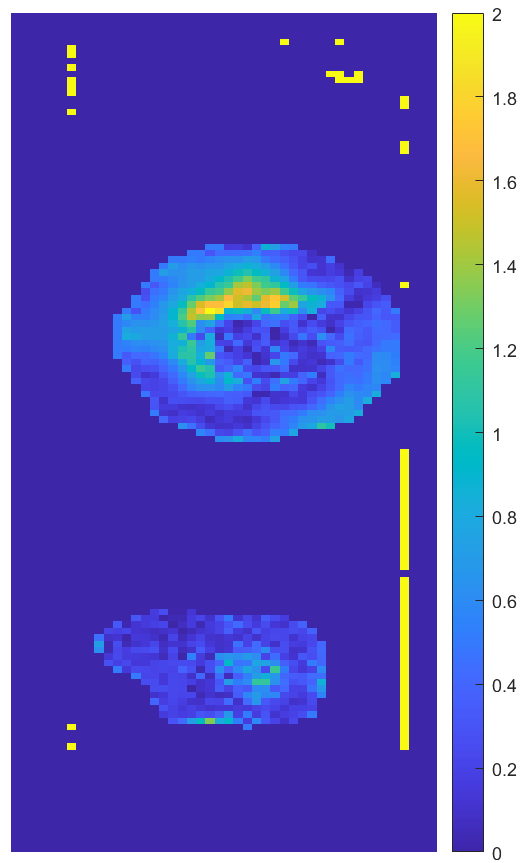 | 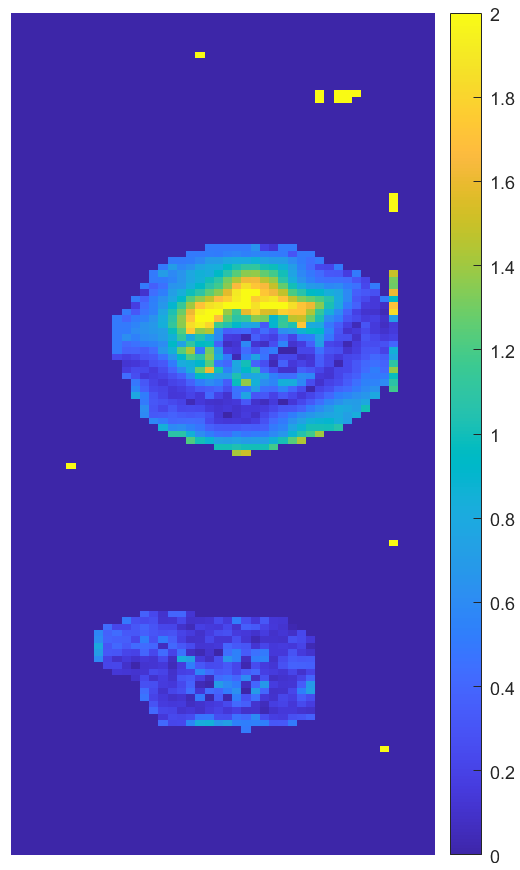 |
| 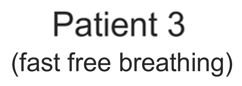 | 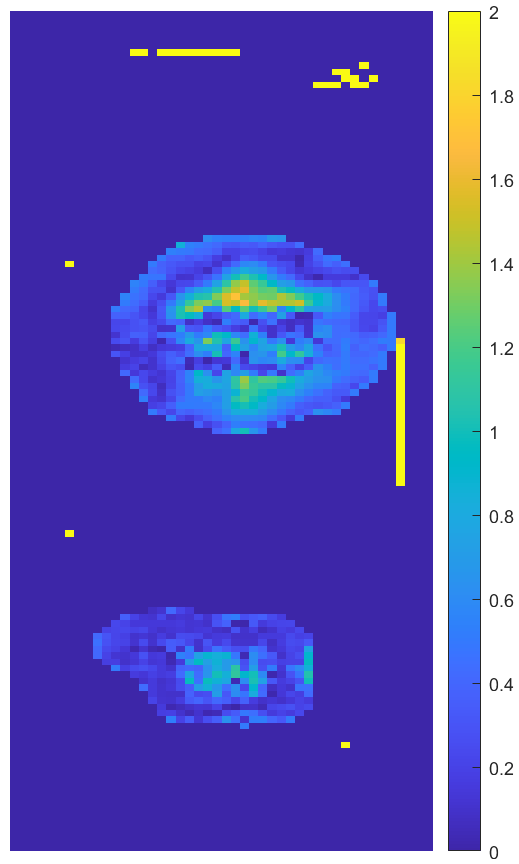 | 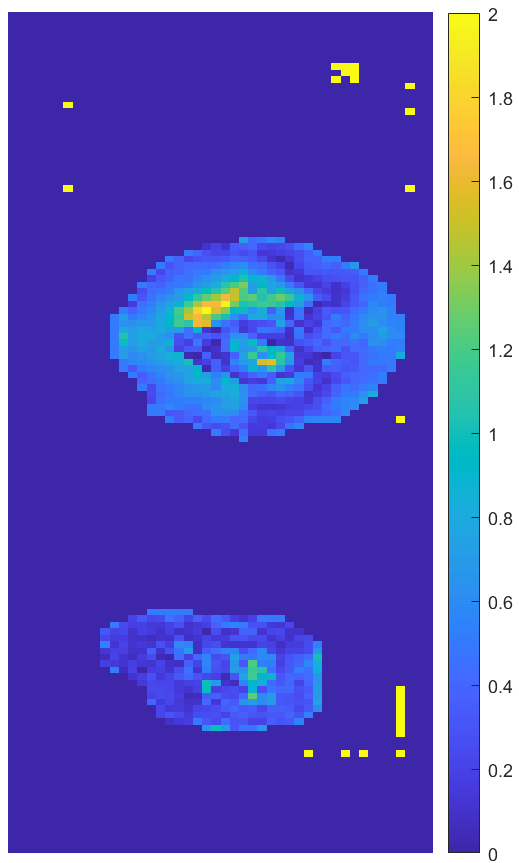 | 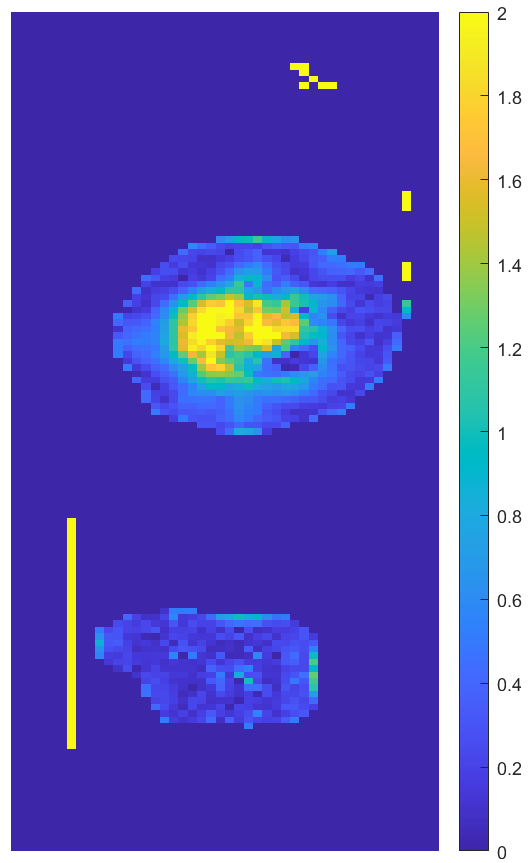 |

(a)

|  | Default (4 fps) | SMT (8 fps) | LDT (8 fps) |
| --- | --- | --- | --- |
| 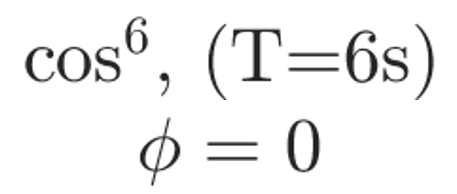 | 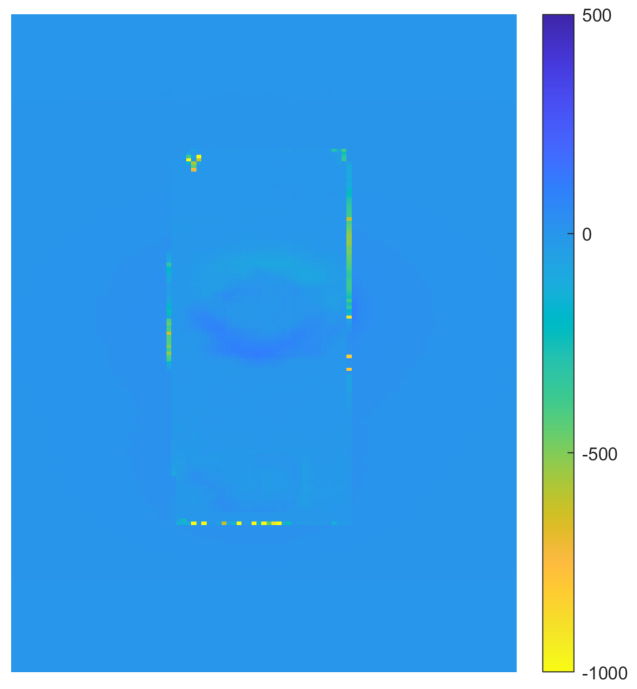 | 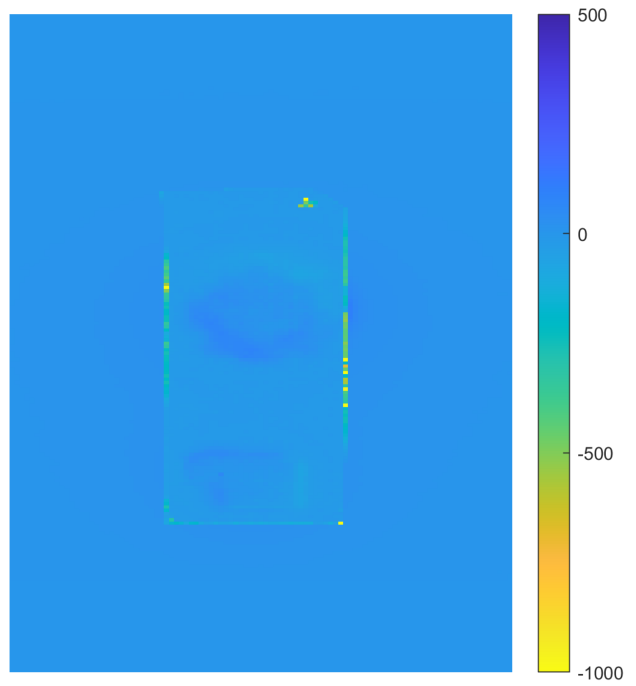 | 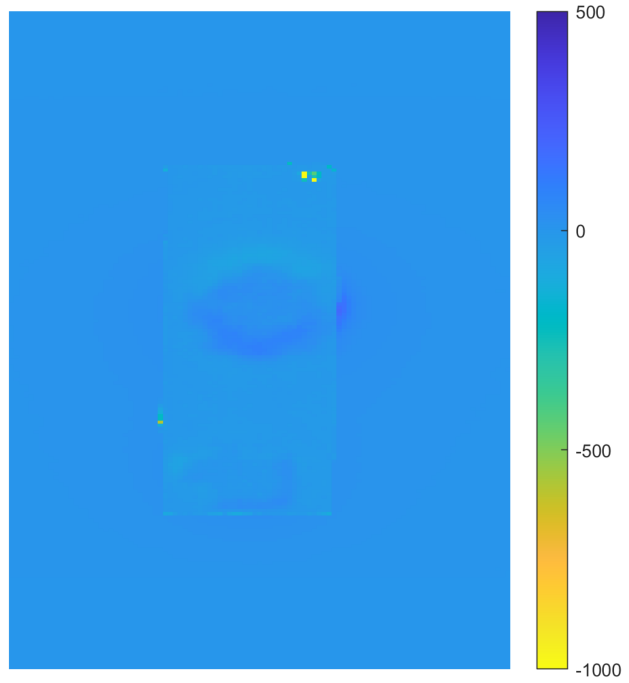 |
| 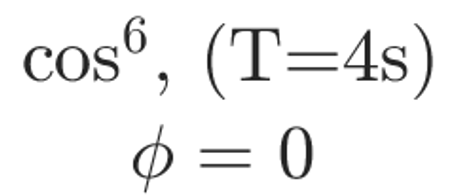 | 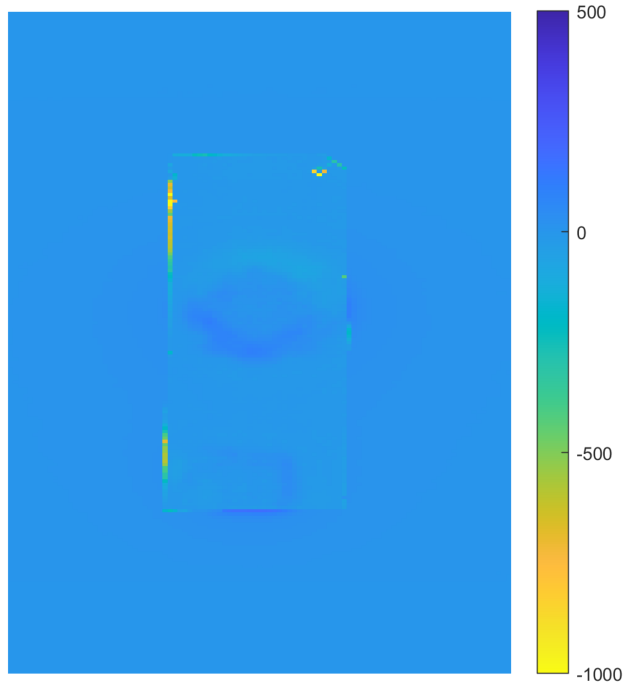 | 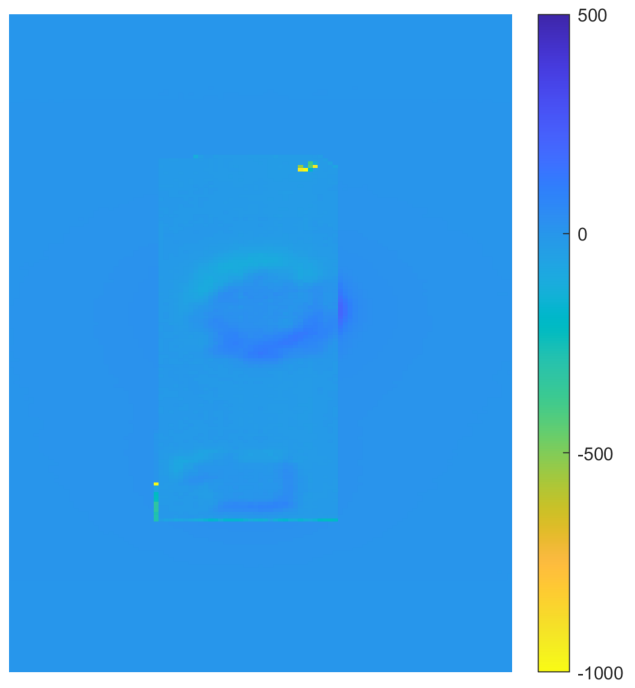 | 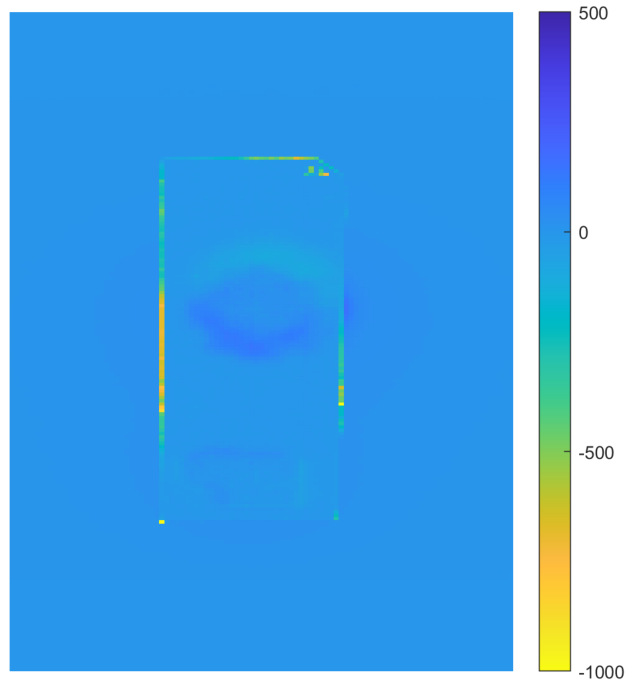 |
| 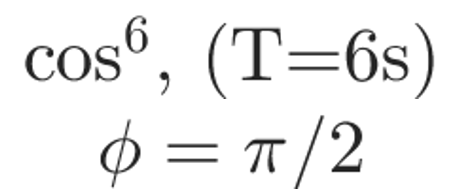 | 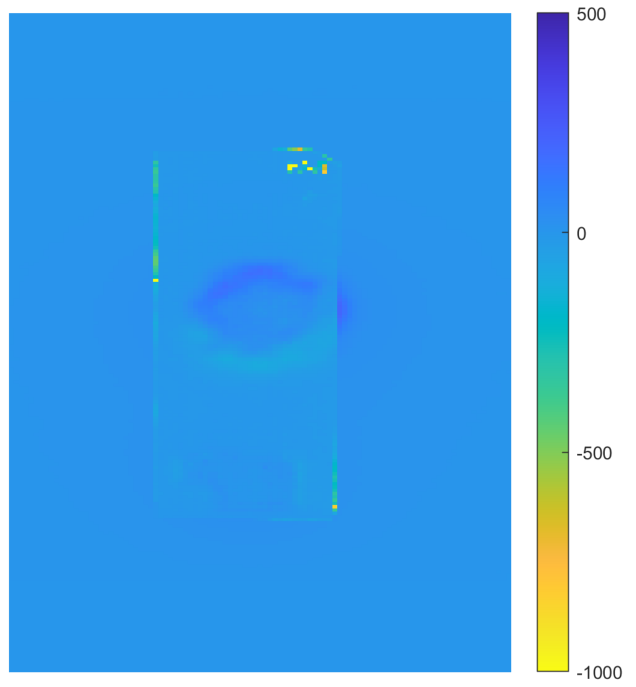 | 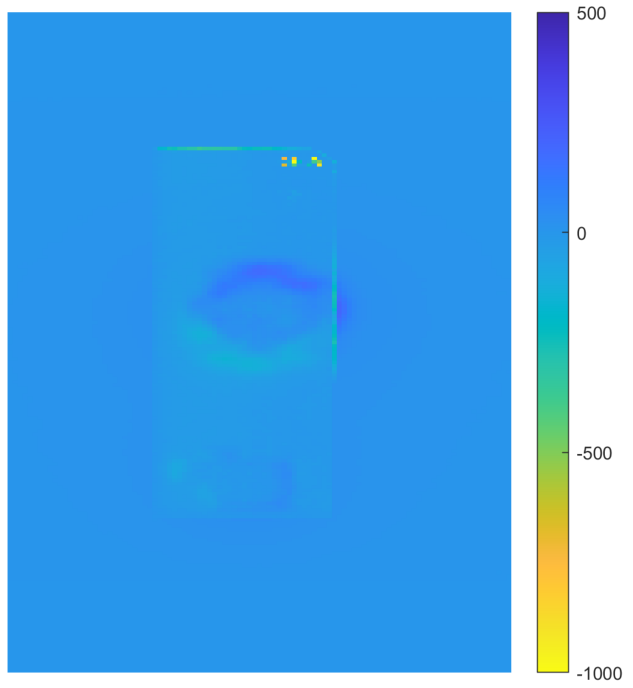 | 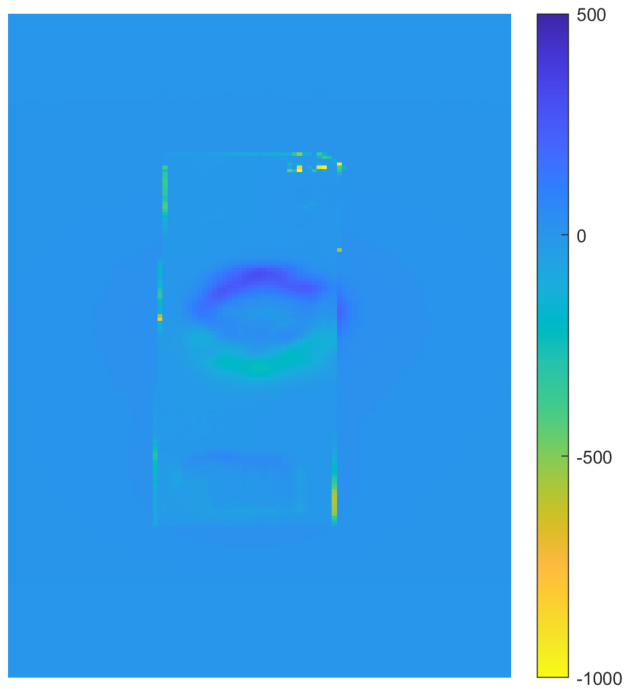 |
| 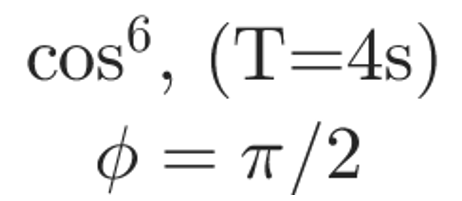 | 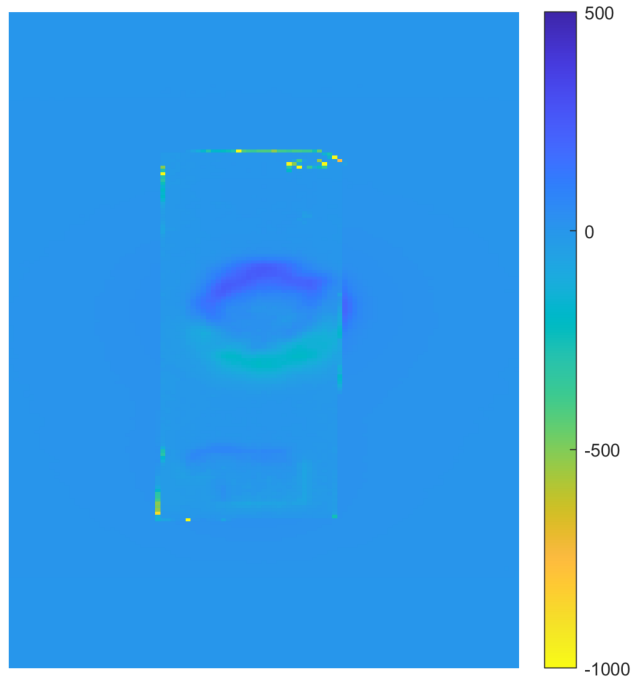 | 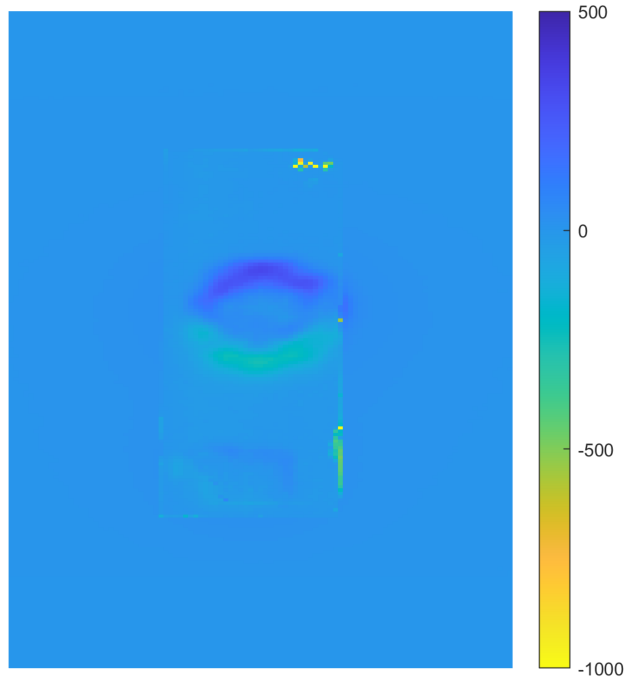 | 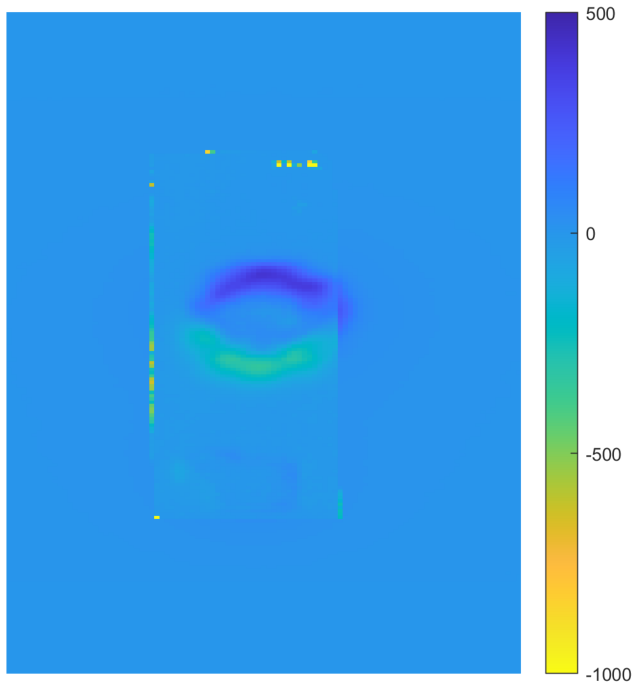 |
| 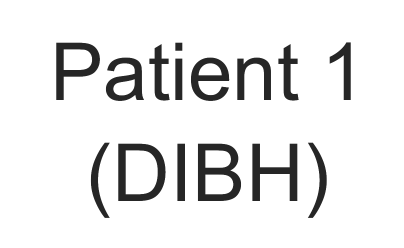 | 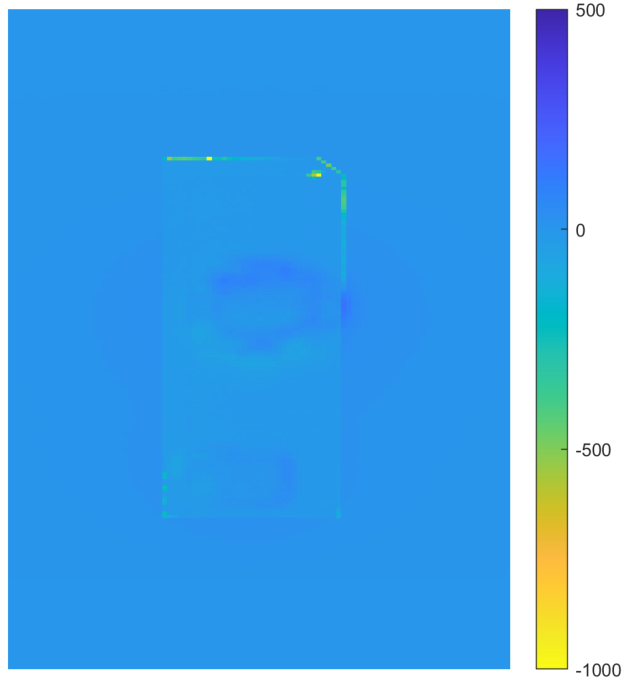 | 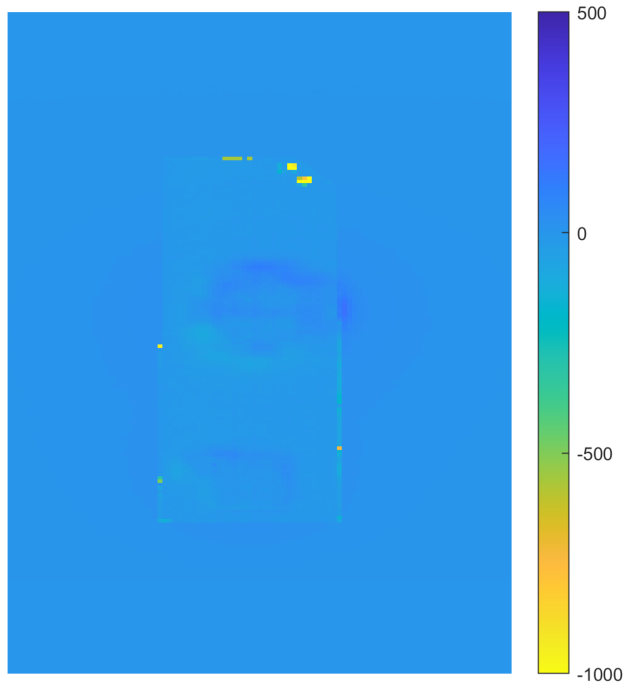 | 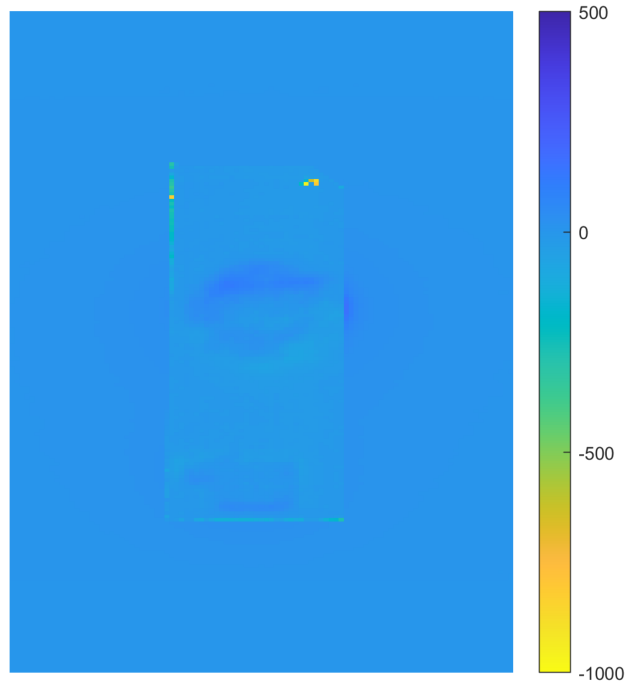 |
| 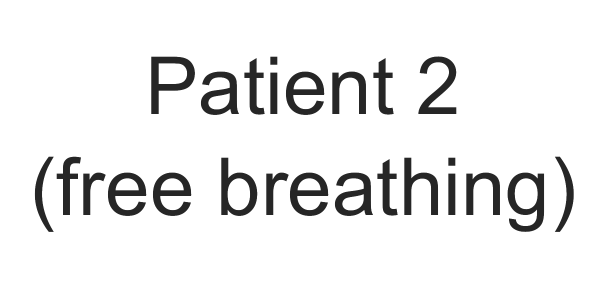 | 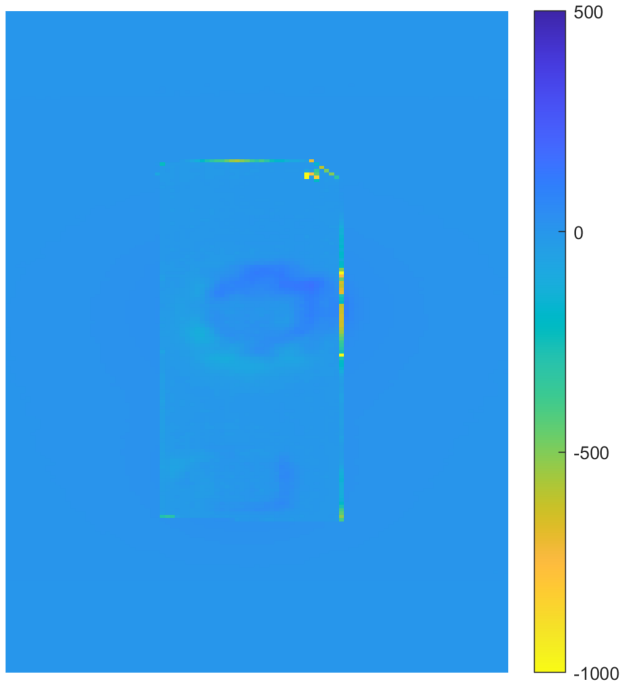 | 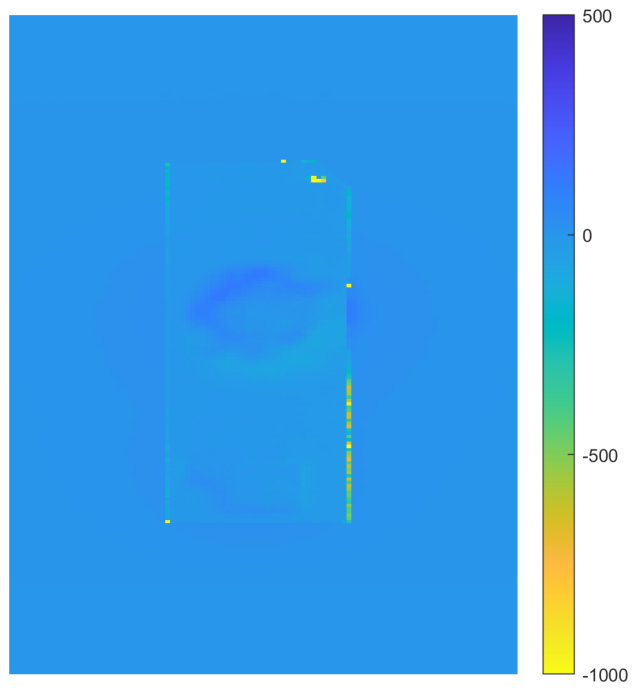 | 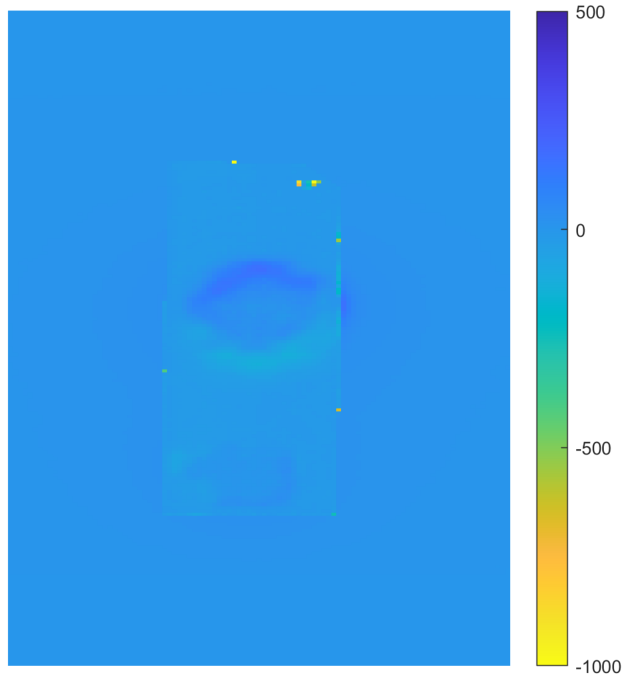 |
| 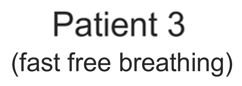 | 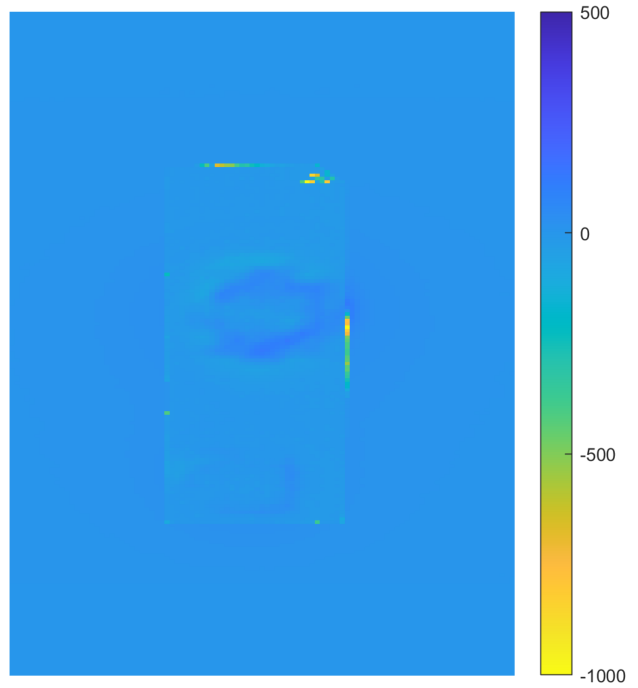 | 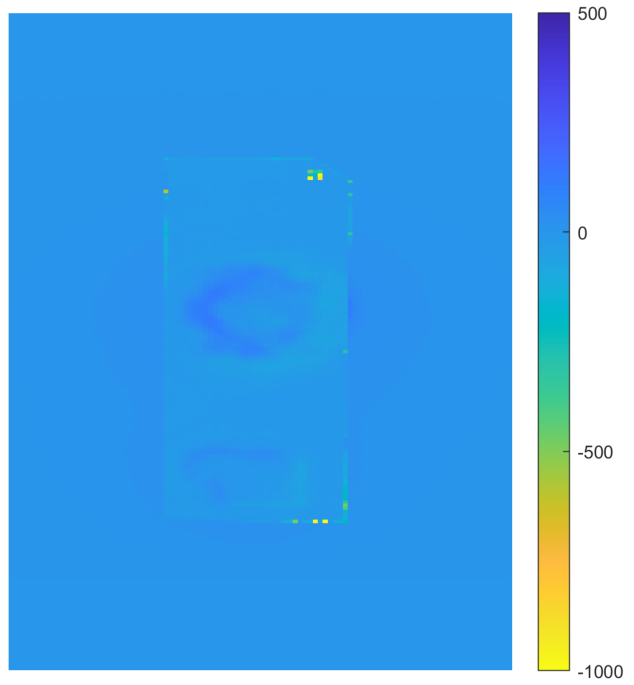 | 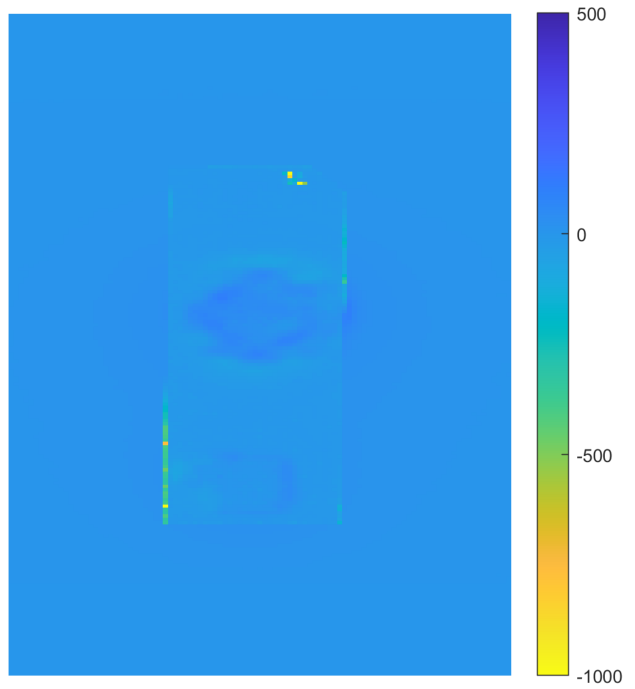 |

(b)

**Supplemental Figure 1:** Gamma maps (a) and dose differentials in cGy (b) for all cases analyzed in this study. We chose a tolerance of 3%, a distance to agreement of 3 mm, and a minimum threshold of 30%. All computed gamma maps are global in the sense that the dose differential is normalized with respect to the maximum planned dose. All dose differential color bars are scaled to the range [-1000, 500] cGy.
