# Supplementary material for: Dosimetric evaluation of respiratory gating on a 0.35‐T magnetic resonance–guided radiotherapy linac
Source: J Appl Clin Med Phys. 2022 Aug 10;23(9):e13666. doi: 10.1002/acm2.13666 (PMC9815517; doi:10.1002/acm2.13666)
Supplement: Supplementary file 2 — Supplementary information [file ACM2-23-e13666-s003.docx]

|  | Default (4 fps) | SMT (8 fps) | LDT (8 fps) |
| --- | --- | --- | --- |
| 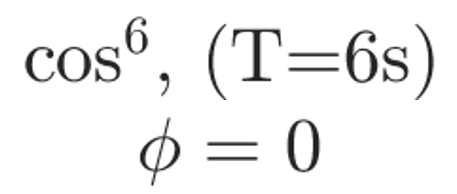 |  |  |  |
| 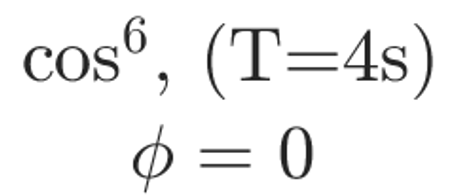 |  |  |  |
| 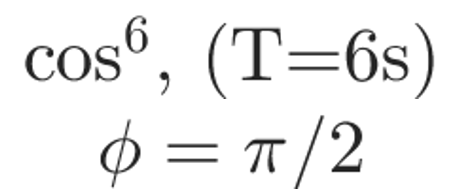 |  |  |  |
| 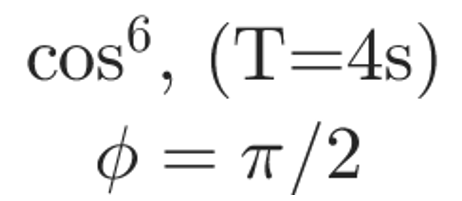 |  |  |  |
| 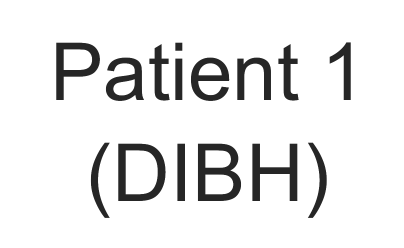 |  |  |  |
| 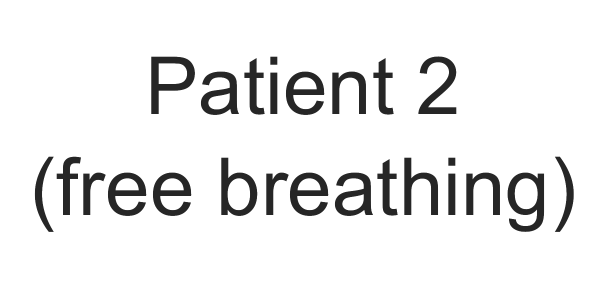 |  |  |  |
| 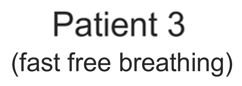 |  |  |  |

**Supplemental Figure 2:** Dose area histograms (DAHs) for all cases analyzed in this study. In order to further assess the dose compactness, we performed a morphological contraction of the original sphere PTV (red) to create GTVs with 3 mm (magenta) and 5 mm (cyan) margins.
