# Supplementary material for: Dosimetric evaluation of respiratory gating on a 0.35‐T magnetic resonance–guided radiotherapy linac
Source: J Appl Clin Med Phys. 2022 Aug 10;23(9):e13666. doi: 10.1002/acm2.13666 (PMC9815517; doi:10.1002/acm2.13666)
Supplement: Supplementary file 3 — Supplementary information [file ACM2-23-e13666-s002.docx]

| **Description** | **3%/3mm** | **2%/2mm** | **1%/1mm** |
| --- | --- | --- | --- |
| 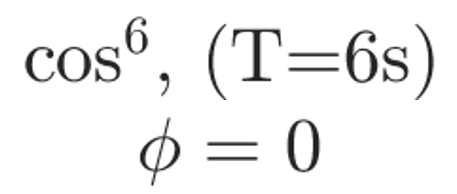  Default | 92.9, 88.2, 90.5 | 63.6, 55.2, 54.7 | 22.3, 13.3, 14.7 |
| 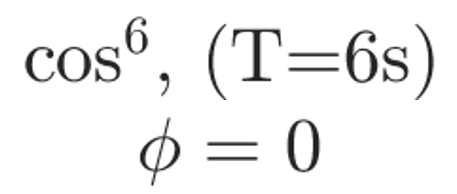  SMT | 90.9, 69.4, 70.4 | 59.4, 33.2, 37.2 | 14.1, 5.5, 8.1 |
| 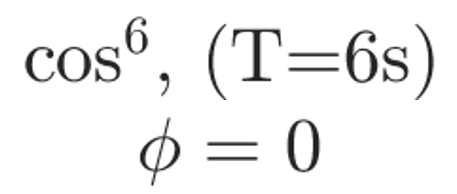  LDT | 71.0, 54.7, 52.1 | 41.8, 27.5, 27.8 | 11.5, 7.6, 6.0 |
|  |  |  |  |
| 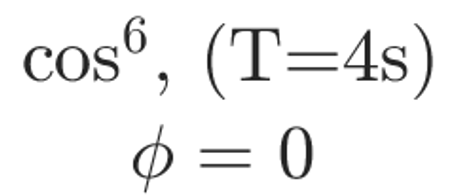  Default | 80.9, 71.9, 74.1 | 47.5, 43.7, 44.0 | 10.8, 7.1, 7.8 |
| 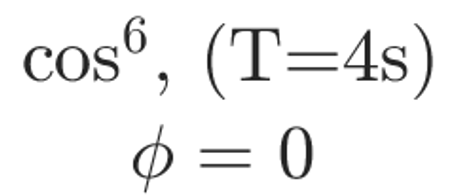  SMT | 79.9, 67.3, 59.2 | 45.5, 33.2, 27.7 | 13.1, 6.4, 7.9 |
| 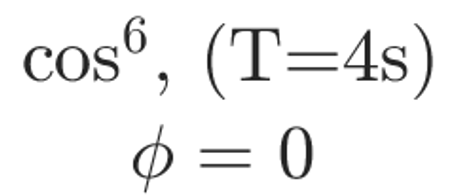  LDT | 58.7, 51.5, 55.3 | 27.7, 24.7, 29.3 | 4.7, 6.8, 4.8 |
|  |  |  |  |
| 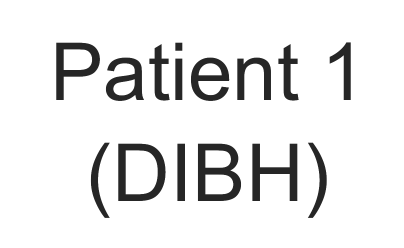  Default | 83.2, 80.8, 80.2 | 59.6, 47.0, 50.3 | 19.7, 9.2, 16.7 |
| 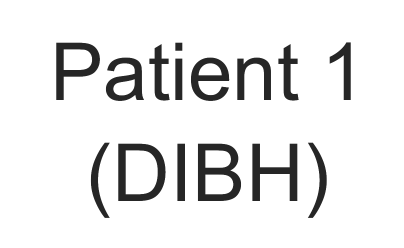  SMT | 87.1, 89.8, 72.3 | 60.5, 67.3, 44.5 | 14.7, 18.5, 8.1 |
| 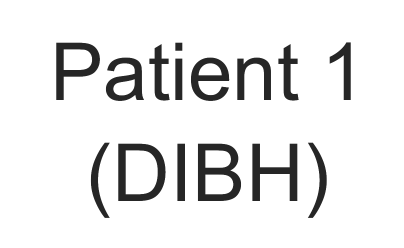  LDT | 74.3, 52.0, 75.2 | 48.7, 31.2, 37.9 | 12.8, 9.6, 8.1 |
|  |  |  |  |
| 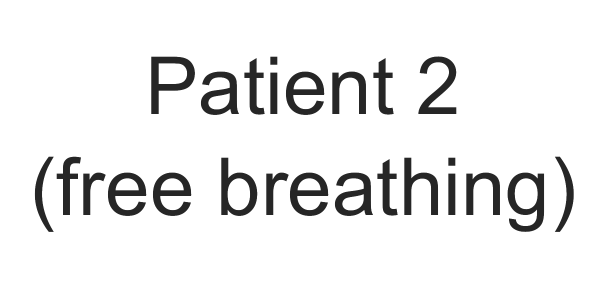  Default | 73.8, 75.8, 59.6, 80.9, 77.4 | 49.7, 53.0, 32.7, 57.5, 55.7 | 15.5, 15.9, 6.0, 19.6, 19.5 |
| 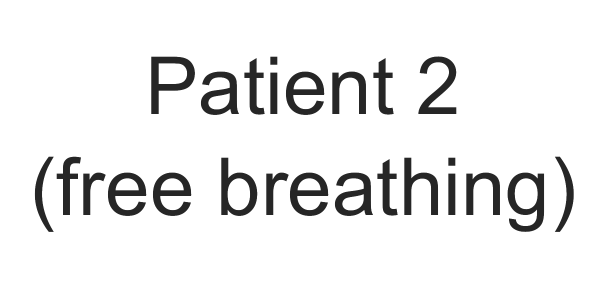  SMT | 77.8, 74.8, 40.1 | 55.5, 47.3, 21.3 | 17.6, 11.8, 3.9 |
| 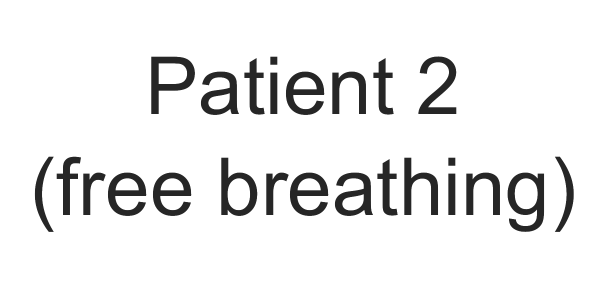  LDT | 53.7, 46.3, 47.3 | 28.0, 24.1, 24.8 | 6.2, 10.7, 4.2 |
|  |  |  |  |
| 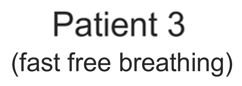  Default | 77.2, 68.1, 57.3 | 52.6, 43.4, 30.4 | 16.8, 14.4, 5.7 |
| 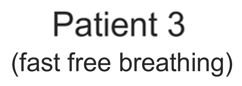  SMT | 84.8, 46.6, 76.2 | 60.7, 21.4, 42.2 | 16.7, 4.1, 8.4 |
| 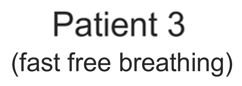  LDT | 63.6, 46.8, 60.5 | 35.8, 24.4, 33.0 | 7.0, 3.4, 4.9 |
|  |  |  |  |
| 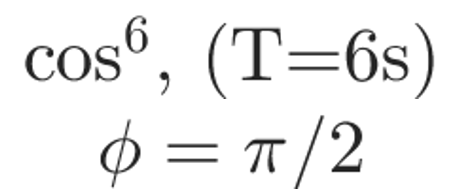  Default | 44.7, 57.9, 50.7 | 22.3, 31.6, 24.2 | 4.5, 6.2, 3.8 |
| 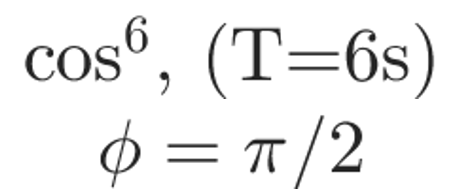  SMT | 57.8, 68.6, 51.5 | 28.5, 42.1, 29.0 | 4.9, 12.6, 7.1 |
| 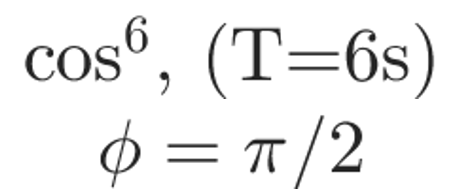  LDT | 41.6, 30.9, 26.9 | 22.4, 13.4, 13.3 | 5.4, 2.6, 2.4 |
|  |  |  |  |
| 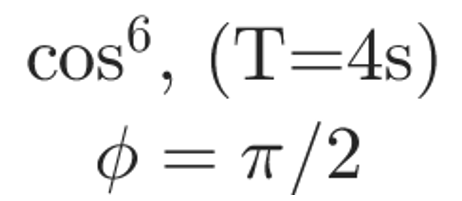  Default | 43.2, 42.4, 45.0 | 21.1, 20.1, 20.9 | 5.6, 5.0, 6.5 |
| 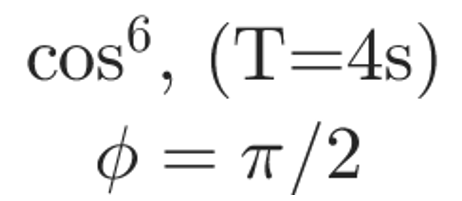  SMT | 22.3, 39.5, 39.2 | 12.1, 23.5, 23.1 | 2.3, 6.5, 8.4 |
| 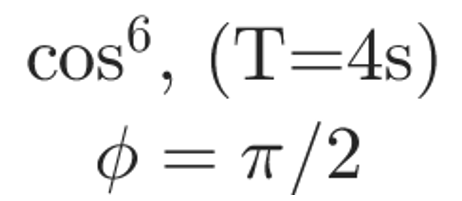  LDT | 17.5, 19.6, 24.0 | 10.0, 12.6, 13.9 | 2.4, 3.9, 3.7 |

Supplemental Table 1: Gamma passing rates for all measurements conducted in this study.
